# Why animals swirl and how they group

**DOI:** 10.1038/s41598-021-99982-7

**Published:** 2021-10-21

**Authors:** Egor E. Nuzhin, Maxim E. Panov, Nikolai V. Brilliantov

**Affiliations:** 1 Skolkovo Institute of Science and Technology, Moscow, Russia 121205; 2 The University of Leicester, University Road, Leicester, LE1 7RH, UK

**Keywords:** Nonlinear phenomena, Machine learning, Applied mathematics, Computer science, Biological physics

## Abstract

We report a possible solution for the long-standing problem of the biological function of swirling motion, when a group of animals orbits a common center of the group. We exploit the hypothesis that learning processes in the nervous system of animals may be modelled by reinforcement learning (RL) and apply it to explain the phenomenon. In contrast to hardly justified models of physical interactions between animals, we propose a small set of rules to be learned by the agents, which results in swirling. The rules are extremely simple and thus applicable to animals with very limited level of information processing. We demonstrate that swirling may be understood in terms of the escort behavior, when an individual animal tries to reside within a certain distance from the swarm center. Moreover, we reveal the biological function of swirling motion: a trained for swirling swarm is by orders of magnitude more resistant to external perturbations, than an untrained one. Using our approach we analyze another class of a coordinated motion of animals—a group locomotion in viscous fluid. On a model example we demonstrate that RL provides an optimal disposition of coherently moving animals with a minimal dissipation of energy.

## Introduction

Swirling is one of the most enigmatic phenomenon of the collective behavior of animals. The circular motion around a common center is observed in large groups of animals at different evolution stages ranging from insects and plant-animal worms to fish. The biological function of such motion is hardly understood and remains under debate till now^1–6^. Moreover, swirling models directly justified by experiments are presently lacking. The existing approaches exploit artificial mechanical forces acting between animals. These interactions have a form of spring-like forces, gravitation-like forces, and forces of Morse potential^7–10^. Certainly, such mechanical forces do not exist but serve to mimic an *intention* of moving animals to change their velocity. This intention is modeled in the form of the second Newton law, which describes the rate of change of animals’ speed. The authors of Ref.^11^ assumed that all particles move with a constant absolute velocity. The directions of the velocities vary in response to the torques originating due to interactions between animals; they mimic the intention of an animal to align its velocity with neighbours. It was assumed that the torques were proportional to the sine of an angle between the direction of motion of an animal and its neighbours from a certain area. The latter was specified by the distance from the animal and the angle of its visual cone (VC)^12,13^, which reflects a limited visual perception of the animal. In some range of parameters, quantifying the VC, swirling patters were observed^11^. Interestingly, swirling emerged only for limited visual perception^11^.

Another set of models stem from the celebrated Vicsek model^14,15^. Here an intention of a moving agent (animal) is explicitly formulated in the form of a *kinematic algorithm*. The algorithm dictates the change of the animals’ velocity. It is assumed that all agents have a constant absolute velocity of varying direction. At each time step the direction is chosen as an average direction of motion of all neighbors, located within a certain distance from the agent. In spite of the simplicity, the model demonstrates a very rich behavior, predicting different modes of collective motion, pattern formation, like in systems of dissipative particles^16,17^ and even phase transitions^9,14,15,18,19^.

Still Vicsek model is too simple to describe swirling motion, which neither arises spontaneously in this model, nor in the presence of a circularly moving leader; the reason for this is a fixed magnitude of the velocity^20^ (note that in the model of Ref.^11^, which may be considered, to some extent, as a continuous-time version of the Vicsek model, swirling emerges under perceptual constraints). Hence there is a challenge to propose an *intention*-based model with a simple kinematic algorithm resulting in swirling. Such an algorithm, based on the a priori knowledge about the agents, should be as realistic as possible. The aim of the present study is to propose a relevant intention-based model of collectively moving agents, which accounts for their perceptional and physical limitations. We expect that such a model will not only predict the emergence of the swirling motion, but also help to understand the biological function of this enigmatic phenomenon.

In a seminal paper^21^, Reynolds mentioned that an adequate model should reflect a limited perception of animals performing coordinated motion (“fish in murky water”, “land animals that see only nearest herdmates”, etc.^21^). Here we analyze the case of limited perception of animals with respect to inter-agent distances, velocities, etc. which are perceived very crudely. A problem of multi-agent connectedness under limited information access has been addressed in^22^, where the existence of algorithms keeping the connectedness has been mathematically proven. This study demonstrates, however, that it is extremely difficult to formulate an explicit action algorithm for systems with a limited information access. Furthermore, the additional constraints, due to the physical limitations of the agents, make such a problem even harder and possibly unsolvable.

Nevertheless, nature finds the solution. Myriads of living beings— insects fish, etc. swirl. It seems extremely improbable that animals follow a sophisticated mathematical algorithm imprinted in their genes. More reasonable is to assume that animals learn to adopt their velocity—both the magnitude and the direction, by trial-and-error method, receiving a reward for a correct action and some form of punishment for an incorrect one. The reward and punishment are regulated by internal chemical processes in animals’ organisms. Hence, the most plausible assumption is that the response to an action (reward or punishment) and the action variability are the fundamental features of animals, imprinted in their genes. This is our main hypothesis, see also the discussion below.

Once we accept such a hypothesis we immediately recognize that the trial-and-error method, supplemented by a reward and penalty is in the heart of the reinforcement learning (RL)—one of the most powerful tools of machine learning (ML) techniques. This method has been successfully exploited for various transport problems of active particles^23–25^, including learning to flock^26^. The application of ML to communication problems (also animal communications) has also demonstrated its efficiency^27–32^. Moreover, the RL applied to biological systems gives a new insight into reward-based learning processes^33^ and, as we show below, helps to reveal the biological function of swirling. In recent studies^34,35^, the RL has been exploited to solve the rendezvous and pursuit-evasion problem for communicating agents possessing only local (limited) information. The inverse RL, which allows to get an individual strategy of the agents yielding the observed collective behavior, was also proposed^35^.

In the present article, we apply the RL approach to describe a collective motion in a swarm under constraints, which reflect a very limited perception and physical limitations of the agents, as dictated by their biological nature. Instead of applying the inverse RL, as suggested in^34,35^, we explicitly consider different individual action rules, that may steam from very limited abilities of the information processing of the animals. This reveals an interesting connection between the agents’ strategy and informational completeness and/or their physical limitations.

As it follows from our analysis, the swirling motion may be understood as a specific form of an escort behavior, when an individual animal tries to remain within a certain distance from a swarm center. Therefore, to illustrate the basic ideas we start from the most simple escort problem, when only a few (in our case—a pair) of animals are involved. Such an escort behavior is observed mainly for mammalians^6,36^ with a high information-processing abilities. For illustrative purposes, however, we consider the escort of animals with different levels of information processing.

The simplest one-to-one escort behavior may be formulated as follows: One animal—the ”follower”, tries to reside within a certain interval of distances from another, independently moving animal—the ”leader”. We demonstrate that depending on the degree of awareness and physical abilities, the follower may choose very different strategies. Some of these strategies look rather astonishing. Next we apply the same methodology for the collective motion, supplementing the rules of one-to-one escort motion by a few rules of collective motion in a large group of animals. We show that the swirling motion arises spontaneously and persists. Then we apply random external perturbation to a swarm and observe that the group of animals, “trained” to perform swirling, demonstrates much stronger resistance to the perturbations, than a non-trained group. This justifies our conjecture about the biological function of the swirling motion (note, that the dynamic programming (see e.g.^37^) may be an alternative way to to find an algorithm yielding swirling motion under information-processing constraints. In application to living beings, however, the policy gradient RL approach seem to be more adequate, as it mimics natural learning processes).

To check whether the RL can find the best strategy, we investigate the optimal locomotion of a group of animals coherently moving in viscous fluid; they also experience physical interactions through the media. The optimal locomotion strategy may be found in this case from a straightforward solution of the coupled equations of motion for the agents. We demonstrate that the RL correctly reproduces all the results of the direct optimization.

## Results

### One-to-one escort strategies

**Figure 1:**
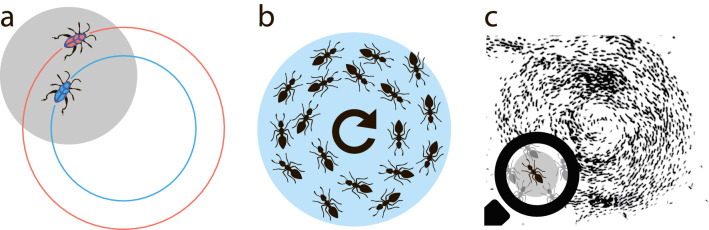
(**a**) Illustration of the escort problem—the leader moves independently on the outer circle, while the follower tries to reside within the comfort zone (shown shaded). (**b**) Illustration of the swirling motion in a swarm. It emerges spontaneously. The comfort zone w.r.t. the center of the swarm is shown (shaded). (**c**) Sketch of the swirling motion in a large colony of insects (ants); the individual comport zone is zoomed. Similar patterns are observed in nature, see e.g.^38^.

In the one-to-one escort a follower tries to reside within some range of distances from an independently moving leader. As we wish to model rather simple animals, we assume a very basic level of their perception. For instance, it is hard to believe that such simple beings can perceive an exact distance between themselves and other objects. At the same time, it is natural to assume the ability of a simpler perception—whether they reside within some distance range from one another. We call this interval of distances, which can be rather large, a “comfort zone”, see Fig. 1a. The notion of the comfort zone will be later applied for swirling motion, see Fig. 1b and c.

Some additional information is needed to construct a trajectory of the follower (in what follows also the “agent”). We assume that the follower can perceive its own direction of motion and this of the leader and can also distinguish between approaching and moving off objects. Next, we consider three different scenarios: (A) The follower can perceive its absolute velocity, and there is no limitation for its acceleration; (B) the follower can perceive its absolute velocity and its acceleration is limited and (C) the follower does not perceive its absolute velocity but can perceive its acceleration, which is limited from above. The last scenario is the most realistic. Indeed, it is not easy to measure the velocity, however, even primitive beings can perceive the direction of motion and their acceleration, trough muscle efforts or other biological mechanisms (even plants “know” the direction of gravitation acceleration). The underlying physiology dictates the limits for the animals’ acceleration.

Leaving the mathematical and algorithmic detail for the section Methods and Supplementary Material (SM), we discuss here the general ideas—how to implement the RL to the addressed problem. In the heart of the RL is the reward—the function of an agent action in a given system state^33,39^. If the action brings the agent closer to the aim, the reward is positive, otherwise—negative. This is the same as the positive and negative stimuli in the nervous system of a living being. The efficiency of the action policy, is characterized by the average of the sum of all rewards (positive and negative) at all actions. The neural network is trained to choose the action policy that maximizes the reward and reaches in this way the desired goal. We wish to stress that the neural network and the according policy is associated with an individual agent. Moreover, we hypothesize that our RL-based model of the agent actions mimics the most prominent features of the real informational processes which determine the behavior of living beings. We investigate different trajectories of a leader—circle, ellipse, eight-curve, spiral, and triangle, which is not a smooth curve. For all these trajectories, the RL managed to train the network to develop successful strategies for the follower.

**Figure 2:**
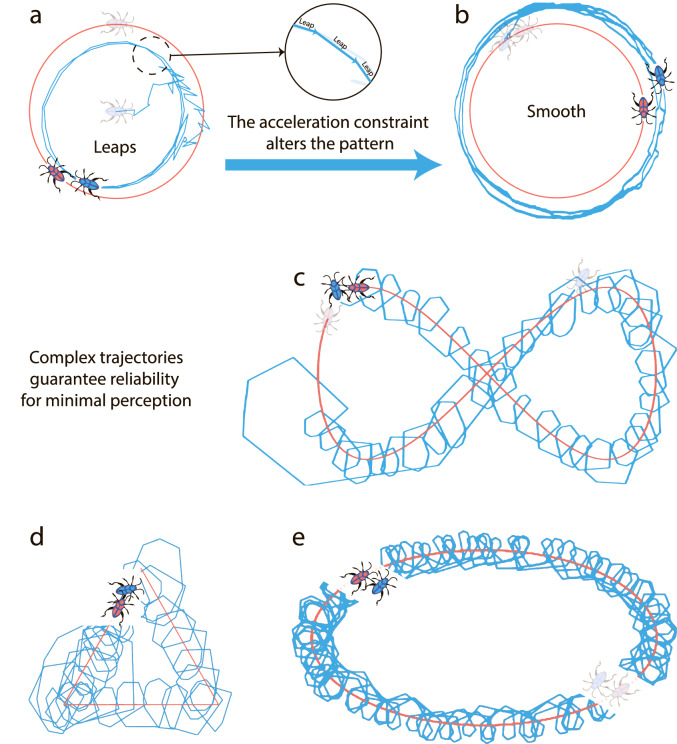
(**a**) The typical trajectory of the follower after the training for A-scenario of escort—the follower can perceive its absolute velocity without a limitation for the acceleration. Note the “frog-strategy” of the follower (see the text). (**b**) The typical trajectory of the follower after the training for B-Scenario of escort—the follower can perceive its absolute velocity and its acceleration is limited. The follower moves on a relatively smooth trajectory. (**c**–**e**) The typical trajectory of the follower after the training for C-scenario of escort—the follower can perceive only its acceleration and the acceleration is limited. The trained follower moves on wrapping trajectories for all trajectories of the leader—circular (not shown), eight-curve (**c**), triangular (**d**) and elliptic (**e**).

The results of the application of the RL to the escort problem are presented in Fig. 2. It is interesting to note that the optimal strategy drastically depends on the available information and physical limitations. Furthermore, the shape of the optimal trajectories of the follower is not necessarily smooth and sometimes looks very astonishing. For the case of “abundant” information, when the velocity of the follower is known, and there are no physical limitations (A-scenario), the follower reaches first the target distance to the leader and then applies the “frog strategy” of successive jumps: It waits until the leader moves far away and makes a long jump. In the new position, it waits again and then jumps, and so on, Fig. 2a. Noteworthy, the jerkily motion with non-smooth trajectories is not related to the discontinuity of the step-function, involved in the estimate of the reward. It persists for other smooth functions, see SM for more detail. When the limitation of the acceleration is imposed, but the agent still perceive its velocity (B-scenario) the follower changes the strategy and moves on smooth trajectories, Fig. 2b.

For the most realistic, C-scenario, when only acceleration is known (which is also limited), the follower starts to use the surprising wrapping trajectories, independently on the shape of the leader trajectory, Fig. 2c–e. In this way it is guaranteed that the follower always remains within a “comfort zone”, even if the information of the velocity and the distance to the leader is lacking. We have also considered an additional energy-saving condition for the C-scenario of the escort. The resulting optimized trajectory remains wrapping, although the wrapping circles become much smaller. The appearance of the curled trajectories for the most realistic C-scenario gives us a hint that these trajectories can transform into swirling for a multi-agents problem.

### The emergence of swirling

Now we address the collective motion (see Methods for mathematical and algorithmic details). As in Ref.^34^ we assume that all animals (also “agents” here) are identical (that is, we do not have leader($\mathbf{s}$) and follower($\mathbf{s}$)). and follow the same action policy. We also assume that any information about an agent is not transmitted to any other agent. That is, $N$ animals learn simultaneously and independently, receiving the reward individually. We believe that the application of the individual training describes more adequately the studied phenomenon and prevents a spurious information exchange between the agents.

To model the collective behavior, we supplement the individual perception rules, as for the escort problem by the collective rules. Namely the following information may be perceived and processed by an agent in a swarm: (i) whether the closest agent approaches or moves off; (ii) whether the closest agent is within a comfort zone; (iii) the direction of motion of the closest neighbor and of the self-motion; (iv) the acceleration of the agent, i.e., the exerted by the agent force; (v) whether the agent approaches or moves off from the center of a group; (vi) the direction of the entire group velocity; (vii) whether the agent resides within a target distance from the group center, which corresponds to the “comfort zone” with respect to (w.r.t.) the group center.

**Figure 3:**
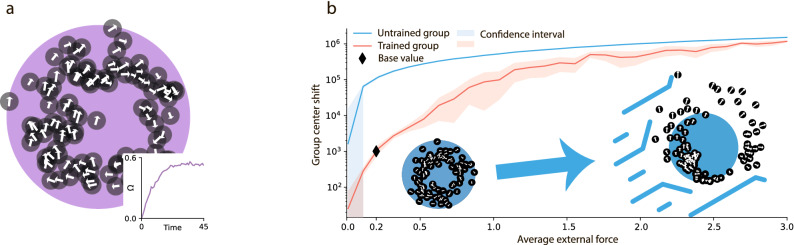
(**a**) Swirling in a swarm of $100$ agents. The large violet circle describes the target distance to the group center, white arrows indicate the speed direction, and the small shaded circles are the agents’ comfort zones. The center of the swarm moves with a constant velocity. Inset: Time dependence of the average angular velocity $\Omega$ of the swarm (see Sect. Methods for the definition). $\Omega$ evolves from zero to non-zero steady-state value. (**b**) The dependence of the shift of a swarm center in the direction of the applied force as a function of the average external force. The results are shown for swarms trained for swirling and untrained swarms. The trained swarm (with swirling) can resist the external force up to $100$ times more efficiently than the untrained one. The resistance of the trained swarm fades for very large external forces when they become comparable to the the maximal force that an agent can exert. The base value marker indicates the reference value of the average force used in SM.

Note that while the rules (i)–(iv) describe the “one-to-one” escort motion, the rules (v)–(vii) describe the behavior in a group. This essentially corresponds to the escort of the group center by an individual animal. Indeed, the reward is given when an agent resides within the comfort zones, both w.r.t. to its nearest neighbor, as well as w.r.t. to the swarm center; otherwise, the agent is penalized. We wish to stress that animals possess a very fuzzy knowledge about the location of their neighbours and the swarm center – they perceive only whether they reside in the according comfort zone, which may be rather large. Also note that we assume that all animals in the swarm are located within the perception zone of each agent, that is, at distances which allow to perceive them. The latter is analogous to the perception circle of Vicsek model^14,15^ and we apply metric, not topological (see e.g.^40,41^) distances between the agents. Here we hypothesize that the rules (i)–(vii) have been imprinted in animals genes by evolution; they motivate all animals in a group to move in a way that fulfills the requested criteria. The agents choose an appropriate policy using (very limited) information at hand. We observe that starting with very different initial conditions, swirling motion of a swarm spontaneously emerges, see Fig. 3a. The swirling around a swarm center is commonly accompanied by a linear motion of the swarm as a whole. The swirling motion may be quantified by the average angular velocity of particles orbiting their common center of mass $\Omega$ (see the next Sect. Methods for the definition). As it may be seen from Fig. 3a, initially $\Omega =0$. In the course of time the swarm self-synchronizes and a steady swirling with non-zero average angular velocity arises and persists.

Note that the condition of swirling—the whole swarm should reside within the perception zone of each animal, implies the size limitations for the swirling group. It should not be too large, which agrees with the conclusion of^42^, where a fish school model, derived from experimental data^43^ has been studied. If the condition, that the whole group resides within the perception zone in violated, swirling is either lacking, $\Omega =0$, or emerges in a subgroup, see Sect. “Algorithms of motion in a swarm and emergence of swirling” for more detail. An untrained swarm, where the actions of the agents do not depend on their states simply disintegrates as disordered Vicsek flocks^14,15^.

### The biological function of the swirling motion

Is it possible to understand the biological function of the swirling motion in swarms? We assume that the swirling helps to resist the external forces, which may jeopardize animals. For instance, it can be a wind that may blow an insects’ swarm far from their inhabitation. To prove this conjecture, we conducted the following experiments. We incorporated an additional external force into the environment and measured the shift of the swarm center in the direction of the external force. During the training, we changed the direction of the external force with a uniform angular distribution. We also modulated the force strength with some periodicity, applying a stretched exponential distribution, widely used to describe natural phenomena, see e.g.^44,45^ and Sect. Methods for detail. We performed the averaging of the swarm shift (in the direction of the force) for different values of the average force. For each average force, we repeat our experiments ten times. We check that varying the parameters of the force distribution does not change the qualitative behavior of the system. Moreover, the behavior of the system persist when the size of the comfort zone or a number of agent in a swarm varies. More details are given in SM.

In Fig. 3b we plot the dependence of the shift of a swarm center on the average external force for the basic version of the stretched exponential distribution (see Sect. Methods); the results for the general case may be found in SM. We compare two groups of agents: The one has been trained to swirl, as described in the previous section, the other—untrained. As it may be seen from the figure, the group that performs swirling resists up to $100$ times more efficiently than that without the swirling. The resistance fades for very strong external forces, comparable with the maximal force which may be exerted by an agent. Hence we come to an important, although surprising conclusion: The intention to move around a swarm center results in extremely high resistance to external perturbations. It seems astonishing that such a simple strategy helps living beings to cope with a hostile environment. In other words, we conclude that the enigmatic swirling motion is not at all a random arrangement or a behaviorial error of a group of animals. In contrast, it plays a crucial role in their survival. Certainly, animals with a complex organization can learn more efficient strategies, but for very simple beasts, this strategy may be the most reliable to resist the environmental threats.

### Optimal swarm locomotion

As we have illustrated above, the RL allows modeling complicated coordinated motion of swarms where the agents receive and process very restricted information about themselves and the system as a whole. Moreover, the RL allows to model systems without formulation of an explicit action strategy—the action policy is developed through the training with the use of an appropriately chosen reward function. Since the explicit action strategy was lacking for the case of swirling motion, it would be worth to check whether the RL provides a truly optimal strategy. Therefore we consider a problem where the optimal strategy is known beforehand and prove that the RL does finds this strategy. Namely, we analyze an optimal locomotion of a group of animals coherently moving in viscous medium. The total energy dissipated by the moving group sensitively depends on the mutual disposition of the agents. The specific energy dissipation per an animal in a group may be significantly smaller than the dissipation of a solely moving agent. That’s why the migrating birds form flocks and cyclists form a compact group in a cycle race—this helps to save a lot of energy. Here we demonstrate that the RL applied to the locomotion problem of a swarm, indeed, yields the optimal disposition of the swarm members.

**Figure 4:**
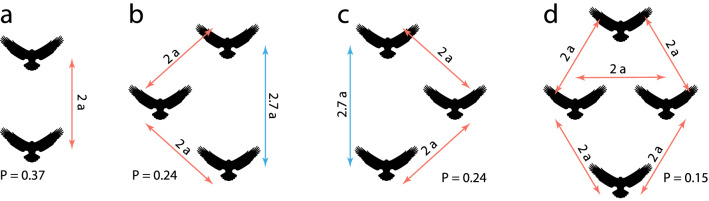
Optimal configurations for the group of two, three, and four agents moving with the same velocity (see SM for the optimal configurations of larger swarms). The results are obtained by the direct minimization of the total dissipation power (see Methods). The average dissipated power per agent is indicated in units of the power dissipated by a solely moving agent. Note that optimal configuration for the triplet has two symmetric realizations. Also note that the larger the swarm, the smaller the specific dissipation.

Realistic modelling of a flock of birds is extremely difficult due to complicated form of birds and a complex flux structure in the air surrounding birds. Moreover, birds fly at high Reynolds numbers. Therefore, to illustrate the concept, we consider a simplified model, where a swarm is comprised of spherical agents moving with velocities corresponding to low Reynolds numbers. These constraints make the model tractable. Indeed, for low Reynolds numbers, there exists an analytical theory for the forces acting between spherical particles moving in viscous fluid (see Methods and SM). Using this theory the optimal configuration may be easily found for any number of agents, as illustrated in Fig. 4.

Next, we check whether the RL is able to find the optimal configurations for the same setup. Namely, we consider a group with a leading animal, which moves with a fixed velocity $\mathbf{v}_{lead}$ and is not affected by other agents of the group. Here we assume that more abundant information may be received and processed. Namely, we assume that the animals have a high perception level, that is, they can perceive: (i) relative positions of the agents and the leader; (ii) whether the nearest neighbor is within the comfort zone; (iii) self-velocity of an agent in the laboratory frame and the relative velocity with respect to other agents; (iv) the force exerted by an agent. The reward function tends to keep agent in their mutual comfort zones and minimize the total dissipation (see Methods and SM for detail).

**Figure 5:**
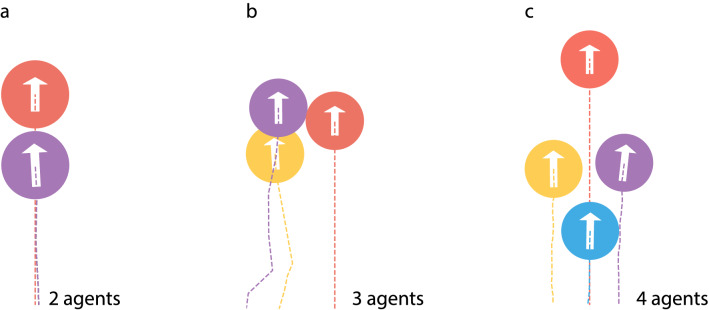
Optimal conformation for the group locomotion, obtained by RL for a system of two (**a**), three (**b**) and four agents (**c**). The red circle depicts the leader comfort zone, and circles of other color show the agent-followers comfort zones. The lines represent trajectories. Note that the configurations found by the RL practically coincides with those, obtained by a direct optimization, Fig. 4.

Fig. 5 illustrates optimal configurations found by the RL, which practically coincides with those obtained by the direct optimization, Fig. 4. These results justify our a’priory trust that the RL manages to find the optimal solution, even if it is not known explicitly.

### Conclusion and outlook

We propose an explanation of the enigmatic phenomenon—swirling motion in large groups of animals at different evolution stage; possibly we reveal its biological function. Our approach is based on the hypothesis that the learning processes in nervous system of animals may be mimicked by the reinforcement learning (RL), with the reward for a beneficial action and punishment for a harmful one. We apply the RL to understand a collective motion of simple animals. They have very limited abilities to perceive and process information about their kinematic states. These limitations are associated with a rather basic level of their organization. We also consider physical limitations caused by the biological nature of the living beings. We formulate a small set of very simple rules which animals strive to follow to conform with the beneficial behavior. We hypothesize that such set of rules is imprinted in animals’ genes.

We assume, that among the main rules of the beneficial behavior in a swarm, is the rule to reside within a certain interval of distances (the comfort zone) from the center of the group and to reside within the comfort zone with respect to other neighbours. Such kind of the individual behavior corresponds to the escort behavior w.r.t. the group center. Therefore, we start with the analysis of the one-to-one escort problem, with a leader and a follower. The leader moves independently on various trajectories and the follower tends to reside within the respective comfort zone. We demonstrate that depending on the information-processing abilities of the follower and its physical limitations, very different escort strategies arise. These include such an amazing behavior as the leap-frog strategy and the strategy of wrapping trajectories.

We demonstrate that the escort strategy of individuals w.r.t the swarm center, results in a spontaneous swirling motion. We observe the emergence of swirling for all studied initial conditions and propose a criterion to quantify it. We assume that in a swirling swarm all animals reside within the perception radius of each other. This puts a natural limiting size for swirling swarms. In other words, in the absence of a limited cone of view of animals, as suggested in Ref.^11^, swirling may emerge in a group which is not too large; this agrees with the conclusion of Ref.^42^. We have also demonstrated that when the size of the system exceeds the perception zone of the agents, swirling of the whole system does not arise. Hence the perception in a limited cone of view as in^11^ is crucial for emergence of swirling in very large groups. This gives a hint for the future extension of the current model to describe swirling in large systems of animals.

Interestingly, we reveal that the swirling motion leads to a dramatic increase of the resistance of a swarm to external perturbations. We believe that this proves the biological function of the swirling—animals swirl to protect themselves in a hostile environment.

We also consider the problem of optimized locomotion of a group of animals moving in viscous fluid. In this case the energy dissipation of a single animal drastically depends on the mutual disposition of the group members. That’s why the organization of the group is so important for coherently moving animals. Using a simplified model, we demonstrate the ability of the RL to find the optimal configuration of a pack. We show that the agents disposition obtained by the RL practically coincide with these found by the direct minimization of the total dissipation, as it follows from the underlying physics. We believe that this result confirms that the RL is able to find the optimal solution, even for the case when such solutions may not be found by traditional mathematical methods.

In our study we use the assumption, that the exploited reward rules have been formed in the course of biological evolution—the process continuing on the evolution time-scale, comprising myriads of species generations. It is governed by random mutations of animals’ genes accompanied by natural selection^46^. This has a strong impact on the evolution of the rewards rules and drives them (by the natural selection) to the most optimal ones for a species survival. The result of the evolution is imprinted in genes of all animals of a species and persists as long as the species exists. In contrast, the training process, has a much sorter time-scale—just a time needed to train an animal. The result of the training is coded in neurons of a particular living being and disappears with its death. Naturally, it is desirable to have a complete model, which describes both, the formation of the reward rules during the biological evolution as well as a training of individual beings. This is however computationally very challenging task and we leave it for future studies.

## Methods

### General principles of the RL

To model an agent (animal) information processing we firstly need to describe the surroundings. That is, we need to formulate the physical properties of the objects and the surrounding media. The respective model is called the “environment”. It describes the system dynamics using the predefined parameters as well as a distribution of these parameters, e.g., the distribution of the initial agents’ positions and velocities. Generally speaking, the environment contains all possible evolution scenarios of the system; its elaboration is in the heart of the approach. Secondly, we need to specify the level of the agents awareness of the environment. The available information about the surroundings refers to an agent state. It contains information about the medium and surrounding objects—their speed, location, etc. It reflects an agent’s understanding of itself as well. Thirdly, we need to define an action space—it determines the way, how the agents interact with the environment. The fourth component is called “policy”; it characterizes the strategy of the agents’ actions. It is quantified by a probability of a certain action at a given state. Finally, the fifth part refers to a reward. The reward is a mechanism to judge, how desirable is the present state as compared to another one. It also assesses a benefit of an action to reach a specific state right now, instead of doing this later.

Once all the above parts are specified, the most computationally intensive part—the learning of the optimal policy, may be performed. Such an approach is called “reinforcement learning” (RL). There are plenty of algorithms to obtain the optimal policy. Below we use the policy gradient algorithm^47^. To summarize, we use the following steps in our analysis:

1. Implement a dynamic simulator of an environment.
2. Identify conditions of the environment available for an agent awareness; define system states.
3. Define a set of actions of an agent. These describe its interactions with the environment and other agents.
4. Define a policy, that is, define an agent actions in response to its state. This is commonly done in a functional form using some parametric class of functions.
5. Identify the motives/goals driving the agents in the form of rewards
6. Run learning to find the optimal policy, corresponding to the optimal set of parameters.

To clarify the analysis we explain in detail the most simple case of scenario C of the escort problem.

*Step 1* The environment comprises the deterministic trajetory of a leader (e.g. a circle) and a dynamic model of a follower. The latter is just a kinematic equation (for a given acceleration, available in the scenario C) solved numerically by the Euler method; initial conditions are set randomly.

*Step 2* The conditions of the environment correspond to the information available for the agent. It is reflected by three vectors and two discrete numbers. The vectors are: the force (acceleration) exerted by the follower and two unit vectors, specifying the velocity direction of the leader and follower. Two discrete numbers (0 or 1) indicate whether the follower resides within the target distance from the leader and whether they approach each other. For the two-dimensional system they form * eight-dimensional* vector $\mathbf{s}$. Hence, the state of the system is defined by the vector $\mathbf{s}$.

*Step 3* For the scenario C the set of actions corresponds to the continuum of two-dimensional forces $\mathbf{F}$ exerted by the follower to achieve the goal (see below). From the physical reasoning (the nature of the agent) the force (or acceleration) is limited, $|\mathbf{F}| \le F_{\mathrm{max}}$.

*Step 4* The policy here (in the scenario C) is the exerted force $\mathbf{F}$ in some state $\mathbf{s}$. In the probabilistic approach, exploited in the RL, it is characterised by the mean ${\varvec{\mu }}$ and variance ${\varvec{\sigma }}^2$. The simplest probabilistic policy, quantifying the probability of the action force $\mathbf{F}$ in a state $\mathbf{s}$, corresponds to the normal distribution (in practice, we exploited a bounded normal distribution, see SM),

1 $$\begin{aligned} {{\mathcal {N}}} \left( {\mathbf{F}} | {{\varvec{\mu }}(\mathbf{s})}, {\varvec{\sigma }}^2(\mathbf{s}) \right) . \end{aligned}$$

Here the vector ${\varvec{\mu }}(\mathbf{s})$ and two-dimensional (diagonal) matrix ${\varvec{\sigma }}(\mathbf{s})$ are functions of a current state $\mathbf{s}$. The very essence of the RL is to find, in the process of learning (see below), the optimal functions ${\varvec{\mu }} (\mathbf{s})$ and ${{\varvec{\sigma }} }(\mathbf{s})$ providing the best actions. To illustrate the idea consider the simplest case when the functions ${\varvec{\mu }} (\mathbf{s})$ and ${{\varvec{\sigma }} }(\mathbf{s})$ are linear functions, say a product of a parameter vector, ${\varvec{\theta }}=\left( {\varvec{\theta }}_{\mu }, {\varvec{\theta }}_{\sigma }\right)$, and a state vector $\mathbf{s}$. That is, ${{\varvec{\mu }}} (\mathbf{s}) = {\varvec{\theta }}_{\mu } \cdot \mathbf{s}$ and ${{\varvec{\sigma }}}(\mathbf{s})= |{\varvec{\theta }}_{\sigma } \cdot \mathbf{s}|$. Hence the goal of the learning is to determine the optimal parameter vector ${\varvec{\theta }}$. The linear functions have however rather limited application as they are not flexible enough. In real practice, more complicated, non-linear functions ${{\varvec{\mu }}_{\varvec{\theta }}} (\mathbf{s})$ and ${{\varvec{\sigma }}_{\varvec{\theta }} }(\mathbf{s})$ are exploited, which makes the approach more accurate and flexible. The most efficient functions for ${{\varvec{\mu }}_{\varvec{\theta }}} (\mathbf{s})$ and ${{\varvec{\sigma }}_{\varvec{\theta }} }(\mathbf{s})$ are realized through neural networks (NNs) used in the present study. The NNs are essentially complicated functions for ${{\varvec{\mu }}_{\varvec{\theta }}} (\mathbf{s})$ and ${{\varvec{\sigma }}_{\varvec{\theta }} }(\mathbf{s})$ operating with parameter matrices, as discussed below. In spite of a surprising efficiency of NNs in many areas of science and industry^48^ their reliability is not rigorously proven yet; still there are solid reasons to trust NNs, see e.g.^49^. We initialize randomly the state $\mathbf{s}$ and the learnable parameters ${\varvec{\theta }}$ and tacitly assume that ${{\varvec{\mu }}_{\varvec{\theta }}} (\mathbf{s})$ and ${{\varvec{\sigma }}_{\varvec{\theta }} }(\mathbf{s})$ converge to the optimal functions.

According to the main theorems of the RL^49^ neither the initial mean nor initial variance are important, since these quantities converge to the optimal ones in the learning process (here we assume that the initialization point belongs to the basin of convergence to the global optimum). In practice, a NN is randomly initialized, yielding some random initial mean and (large) variance, almost independent on a state, see the discussion below.

*Step 5* The agent (the follower in the scenario C) strives to reside within a range of target distances from the leader. It is rewarded (or punished) if the goal is achieved (or not achieved). The reward is quantified by the reward (loss) function. The cumulative reward *R* is the sum of the rewards at each time step; more detail are given below. *R* is defined for some time interval called a ”simulation episode”.

*Step 6* To find the optimal policy implies (for the addressed problem) to determine ${{\varvec{\mu }}_{\varvec{\theta }}} (\mathbf{s})$ and ${{\varvec{\sigma }}_{\varvec{\theta }} }(\mathbf{s})$, or equivalently ${\varvec{\theta }}$ maximizing the average cumulative reward. Here we apply the effective tool—the policy gradient (PG) method. Let us explain the main ideas of the method, omitting technical and mathematical detail, see SM and^47^ for the mathematical rigor. The PG method works as follows: we run *n* simulation episodes, each of the time length *T*, generating the “trajectories” $\left( \mathbf{s}_0, \mathbf{s}_1,\ldots \mathbf{s}_T \right)$ for the episodes. For each, say *k*-th episode we compute a series of immediate rewards $\left( r(\mathbf{s}_0), r(\mathbf{s}_1),\ldots r(\mathbf{s}_T) \right)$. Using the latter quantities and applying the gradient estimator “EPISODIC REINFORCE” (see^50^ for the underlying math), the gradient of the average cumulative reward $\langle R \rangle _k$ with respect to ${\varvec{\theta }}$ may be obtained, see SM for more detail. Next, we update the current parameters ${\varvec{\theta }}$ as:

$$\begin{aligned} {\varvec{\theta }}_{k+1} ={\varvec{\theta }}_{k} + \eta \nabla _{\varvec{\theta }} \langle R \rangle _k, \end{aligned}$$

where the technical coefficient $\eta$ controls the convergence (the learning rate). Then we repeat this procedure for $(k+1)$-th episode with the policy parameters ${\varvec{\theta }}_{k+1}$, and so on. The learning procedure has been realized through deep neural networks discussed below.

Up to now we detailed the application of the RL to the escort scenario C. The scheme does not differ much for other systems. For instance, for the escort scenarios A and B, the role of action plays the velocity (instead of exerted force in C). Correspondingly, the probability distribution, characterising the policy, has the form (1), with the force $\mathbf{F}$ substituted by the velocity $\mathbf{v}$ (for the escort scenario B, however, some restrictions are imposed, see SM).

For multi-agent systems with swirling, or effective locomotion, the policy coincides with the policy of the escort scenario C, which is applied to each agent. The main difference refers to the reward function of an agent. For the latter systems it depends on the states of all other agents, see Sect. Physical background and algorithms for RL of efficient locomotion of a swarm and SM for detail.

### Environment

We start with the random initialization and then continue with the dynamic simulations. Namely, we generate random initial positions and velocities of all agents. For experiments with the additional external force the direction of the force (wind direction) was randomly chosen and remained constant during a simulation episode. The magnitude of the force *f* randomly varied with time, subjected to the specified probability distribution. In our study we exploit the stretched exponential distribution, widely used to model natural phenomena^44,45^:

$$\begin{aligned} P(f \mid f_0,\beta ) = \frac{1}{f_0} \frac{\beta }{\Gamma (1/ \beta )} \, \cdot e^{-(f/f_0)^{\beta }}. \end{aligned}$$

Here $\Gamma (x)$ is the Gamma function, $\beta$ is the stretching exponent and $f_0$ is the scale factor. We mainly use the simplest version with $\beta =1$, which is called exponential distribution. $f_0$ in this case corresponds to the distribution of a mean value. Other distributions from this class with a wide range of $\beta$ have been also tested. This does not change the qualitative behavior of the system, see SM for more detail. To model the evolution of the environment we solve time-discretized equations, where the variables are calculated using their values on the previous time step, applying the action policy. The simulations were repeated from one episode to another, iteratively improving the action policy, in accordance with the learning rules.

### Policy

The action policy has been developed for various types of actions (shortly discussed above for scenario C of the escort problem). All these require continuum random variables. Here we use normally distributed variables with a slight modification, that prevents an occurrence of very large quantities forbidden by the underlying physics, see SM for detail.

Let $\pi _{\varvec{\theta }}({\mathbf{a}}_t \mid {\mathbf{s}}_t)$ be the policy associated with the action ${\mathbf{a}}_t$ at a given state ${\mathbf{s}}_t$ at time $t$, that is $\pi _{\varvec{\theta }}({\mathbf{a}}_t \mid {\mathbf{s}}_t)$ gives the probability density of the action ${\mathbf{a}}_t$ at a state ${\mathbf{s}}_t$. It is parametrized by the set of parameters $\varvec{\theta }$. For the scenario C, discussed above, the policy $\pi _{\varvec{\theta }}({\mathbf{a}}_t \mid {\mathbf{s}}_t)$ corresponds to the normal distribution of action forces (1).

The reward $r_t = r_t({\mathbf{a}}_t,{\mathbf{s}}_t)$ depends on an agent state ${\mathbf{s}}_t$ and its action in this state ${\mathbf{a}}_t$ and quantifies the advantage of the action ${\mathbf{a}}_t$ in the state ${\mathbf{s}}_t$. The quality of the whole process, i.e., the quality of the action policy, may be assessed as the average sum of the successive (discounted) rewards:

$$\begin{aligned} \langle R \rangle _{\pi _{\varvec{\theta }}} = \langle r_{0} + \gamma r_{1} + \gamma ^2 r_{2} + \ldots \rangle _{\pi _{\varvec{\theta }}} = {\mathbb {E}}_{\pi _{\varvec{\theta }}} \left[ \sum _{k=0}^{T} \gamma ^k r_{k} \right] , \end{aligned}$$

where $0< \gamma < 1$ is the discount factor (we use $\gamma = 0.9$), $T$ is the duration of the training episode and the subscript $\pi _{\varvec{\theta }}$ denotes averaging over the policy. Then the optimal policy parameters ${\varvec{\theta }}^*$ maximize the average reward $\langle R \rangle _{\pi _{\varvec{\theta }}}$, that is,

$$\begin{aligned} {\varvec{\theta }}^*= \arg \max _{\varvec{\theta }} \langle R \rangle _{\pi _{\varvec{\theta }}}, \end{aligned}$$

($\arg$ denotes the argument of the function). In practice, the solution of the above equation is performed with the use of the policy gradient (PG)^47^ sketched above and implemented with the neural network, see SM for detail.

### Neural network architectures

**Figure 6:**
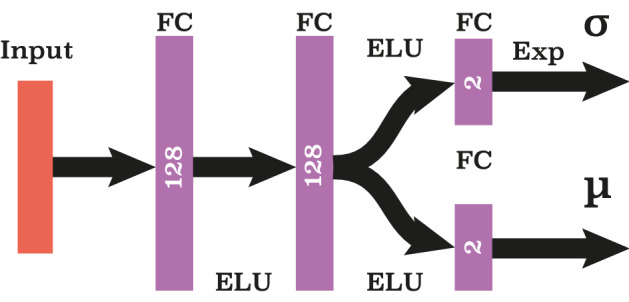
The architecture of the neural network for the action policy.

The policy exploited in our study depends on two sets of parameters associated with the mean and variance of the normal distribution. Since the action space is two-dimensional (the motion occurs in 2D), we have two means and two variances. The number of observed states strongly depends on the problem, however the neural network architecture remains the same. For the action policy this comprises three fully connected layers and two ELU (Exponential Linear Unit) activation functions^51^ between them, see Fig. 6. The exponential activation function is used to avoid negative values of the variance.

The neural network (NN) in our problem is, essentially, the set of actions to construct the functions ${{\varvec{\mu }}_{\varvec{\theta }}} (\mathbf{s})$ and ${{\varvec{\sigma }}_{\varvec{\theta }} }(\mathbf{s})$, which depend on the learning parameters $\varvec{\theta }$ and then optimize them with respect to $\varvec{\theta }$. The learning parameters may be partitioned in two classes—weights matrices ${\mathbf{W}}$ and bias vectors ${\mathbf{b}}$. Below we illustrate the principle of information processing by neural network for C-scenario of the escort problem. The input here is the 8-dimensional state vector ${\mathbf{s}}$. The first layer of the NN is specified by the weights matrix ${\mathbf{W}}_1$ of size $128 \times 8$, bias vector ${\mathbf{b}}_1$ of size 128 and ELU activation function. The output of the first layer ${\mathbf{h}}_1 ({\mathbf{s}})$ reads:

$$\begin{aligned} {\mathbf{h}}_1({\mathbf{s}}) = ELU({\mathbf{W}}_1 \cdot {\mathbf{s}}+{\mathbf{b}}_1). \end{aligned}$$

Here the ELU function, defined as

$$\begin{aligned} ELU(x) = {\left\{ \begin{array}{ll} x &{} \text {if } x > 0, \\ e^x - 1 &{} \text {otherwise}, \end{array}\right. } \end{aligned}$$

is applied, element-wise, yielding the vector ${\mathbf{h}}_1 ({\mathbf{s}})$. The second layer has the similar structure. It is specified by $128 \times 128$, weights matrix ${\mathbf{W}}_2$ and bias vector ${\mathbf{b}}_2$ of size 128, with the same activation function. It yields the second hidden state:

$$\begin{aligned} {\mathbf{h}}_2({\mathbf{s}}) = ELU({\mathbf{W}}_2 \cdot {\mathbf{h}}_1({\mathbf{s}}) +{\mathbf{b}}_2). \end{aligned}$$

The third output layer depends similarly on the second hidden state ${\mathbf{h}}_2({\mathbf{s}})$, but is split into two parts. The first part gives rise to the mean ${\varvec{\mu }}_{\varvec{\theta }}({\mathbf{s}})$ and the second one—to the standard deviation ${\varvec{\sigma }}_{\varvec{\theta }}({\mathbf{s}})$. For C-scenario the policy corresponds to the force exerted by an agent. The mean and standard deviation are two-dimensional vectors for two-dimensional space of actions. The third layer is defined by two weight matrices ${{\mathbf{W}}}_{3}^{\mu }$, ${{\mathbf{W}}}_{3}^{\sigma }$ which have size 128x2, two bias vectors ${\mathbf{b}}_{3}^{\mu }$, ${\mathbf{b}}_{3}^{\sigma }$ of size 2 and one exponential activation function for standard deviation output (since the standard deviation can not be negative). The mean and standard deviation are calculated as follows:

$$\begin{aligned} {\varvec{\mu }}_{\varvec{\theta }}({\mathbf{s}})= & {} {{\mathbf{W}}_{3}^{\mu }} \cdot {\mathbf{h}}_2({\mathbf{s}}) +{\mathbf{b}}_{3}^{\mu }, \\ {\varvec{\sigma }}_{\varvec{\theta }}({\mathbf{s}})= & {} \exp \left[ {\mathbf{W}}_{3}^{\sigma } \cdot {\mathbf{h}}_2({\mathbf{s}}) +{\mathbf{b}}_{3}^{\sigma } \right] . \end{aligned}$$

Once ${\varvec{\mu }}_{\varvec{\theta }}({\mathbf{s}})$ and ${\varvec{\sigma }}_{\varvec{\theta }}({\mathbf{s}})$ are constructed, one can apply the procedure discussed above: Compute the immediate rewards, compute the cumulative reward, apply the PG method and eventually find the optimal parameters $\varvec{\theta }=\varvec{\theta }^*$. However, a natural question arises—how to compute a gradient for such multi-layered structures, which is needed to update the learning parameters? Here we just mention that there exists a special technique, known as backpropagation^49^. Often automatic backpropagation is incorporated into deep learning packages, such as e.g. Pytorch (https://pytorch.org) and Tensor Flow (https://www.tensorflow.org) Python packages.

The parameter matrices $\mathbf{W}_i$ and vectors $\mathbf{b}_i$, with $i=1,2,3$, characterizing the NN were randomly initialized. A simple uniform distribution with the width depending on the size of the matrices/vectors was used, see SM.

### Algorithms of the escort problem

First we consider the escort problem which consists of a single agent-follower moving in an inert two-dimensional space and a leader, moving on a predefined trajectory. The goal of the follower is to pursue the leader, more specifically—to reside within some range of distances from the leader.

Let the leader position be $\mathbf {x}_l$ and the follower—$\mathbf {x}_f$. Then the following information available for the follower reads:

1. Whether the follower resides within a range of distances from the leader:
  $$\begin{aligned} S_d = H\bigl (e_t - \bigl |d_t - \,\Vert \mathbf {x}_{l} - \mathbf {x}_{f}\Vert \,\bigr |\bigr ), \end{aligned}$$
  where $H(x)$ is the Heaviside step function, $d_t$ is the average distance from the leader and $e_t > 0$ specifies the range of acceptable distances.
2. Whether the follower approaches the leader:
  $$\begin{aligned} S_a = H\left( -\frac{d}{dt} \Vert \mathbf {x}_{l} - \mathbf {x}_{f}\Vert \right) . \end{aligned}$$

The goal of the agent is to maximize the reward which is computed by the Algorithm 1. We encode the reward function in the way that the most desirable state is to reside within a range of target distances from the leader. However, if the agent is not close enough, the reward is given for the pursuit of the leader.
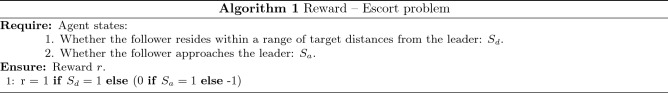


The second important part of the policy refers to the actions which a follower can perform to maximize the reward. We define the leader velocity as $\mathbf {v}_l$ and the follower velocity as $\mathbf {v}_f$. We assume that the follower knows the direction of the leader velocity ${\hat{\mathbf {v}}}_l = \mathbf {v}_l / \Vert \mathbf {v}_l\Vert$ and the direction of its own velocity ${\hat{\mathbf {v}}}_f = \mathbf {v}_f / \Vert \mathbf {v}_f\Vert$. For the scenarios A and B detailed in the Section Results the follower also knows the magnitude of $\mathbf {v}_f$; that is, the agent possesses a complete information about its velocity. Hence, the agent (follower) actions refer to the regulation of its own velocity. In real life, however, the velocity is limited, which implies the limitation of actions that needs to be reflected in the policy. For the scenario B there exist an additional limitation for an agent acceleration, which is also reflected in the policy, see SM.

Here we discuss in detail the most realistic escort scenario C, where the information about the velocity of an agent (follower) is not available, but the agent can control the force, that is, regulate its acceleration. Namely, the agent possesses the following information characterising the state of the environment: (i) the direction of the leader velocity ${\hat{\mathbf {v}}}_l = \mathbf {v}_l / \Vert \mathbf {v}_l\Vert$, (ii) the direction of the follower velocity ${\hat{\mathbf {v}}}_f = \mathbf {v}_f / \Vert \mathbf {v}_f\Vert$ and (iii) the follower force $\mathbf {F}_f$. The available information about the space location has been itemised above. The actions of an agent for the scenario C refer to the regulation of its force, instead of the velocity, as in the scenarios A and B. The force, and thus the acceleration is limited, as in the scenario B.

Noteworthy, without a knowledge of a follower/leader position and a follower/leader absolute velocity, it is extremely difficult (if possible) to obtain an explicit policy of the optimal pursuit. The reinforcement learning allows to construct an efficient stochastic policy for a very limited available information.

### Algorithms of motion in a swarm and emergence of swirling

Similarly to the escort scenario each agent moving in a swarm exploits the available information of its state. We additionally assume that the agents strive to reside not far from the group center and supplement the agent state with extra parameters which specify the desired interval of distances from the group center located at $\mathbf{x}_{gc}$. That is, we use the concept of the target range of distances $d_{t}$ from the group center around $\mathbf{x}_{gc}$. Each agent strives to reside within the radius $d_{cz}$ (i.e. in the comfort zone) from each other. Thus, each *i*th agent in the swarm perceives the following data:

1. Whether the closest neighbour breaks into the comfort zone $d_{cz}$:
  $$\begin{aligned} S_{cz}^i = H\left( d_{cz} - \Vert \mathbf {x}_{i} - \mathbf {x}_{c_i}\Vert \right) , \quad c_i = \arg \min _{j\ne i} \Vert \mathbf {x}_{i} - \mathbf {x}_{j}\Vert , \end{aligned}$$
  where $\mathbf {x}_{c_i}$ is the coordinate of the nearest neighbour and $H(x)$ is the Heaviside step function.
2. Whether the agent approaches the closest agent:
  $$\begin{aligned} S_{ca}^i = H\left( -\frac{d}{dt} \Vert \mathbf {x}_{i} - \mathbf {x}_{c_i}\Vert \right) . \end{aligned}$$
3. Whether the agent resides within a target distance $d_{t}$ to the group center $\mathbf{x}_{gc}$:
  $$\begin{aligned} S_{gc}^i = H\bigg (d_{t} - \Vert \mathbf {x}_{i} -\mathbf{x}_{gc}\Vert \bigg ). \end{aligned}$$
4. Whether the agent approaches the group center:
  $$\begin{aligned} S_{ga}^i = H\left( -\frac{d}{dt} \Vert \mathbf {x}_i -\mathbf{x}_{gc}\Vert \right) , \quad \mathbf{x}_{gc} = \frac{1}{N} \sum \limits _{i = 1}^{N} \mathbf {x}_i, \end{aligned}$$
  where $N$ is a number of the agents.

Based on these data we define the reward, see the Algorithm 2. It encodes our assumptions about an agent striving. The most desirable agent states are realized when the agent resides within a target distance to the group center. However, the agent is penalized if someone brakes into its comfort zone. Finally, if the agent is not within a target distance, we reward a pursuit of the group center.

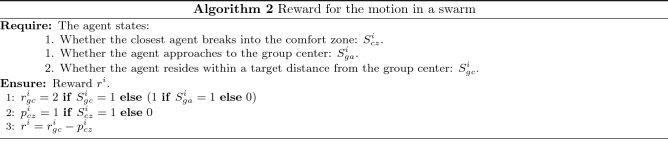



Additionally, each agents knows the direction of the closest agent velocity ${\hat{\mathbf {v}}}_{c_i} = \mathbf {v}_{c_i} / \Vert \mathbf {v}_{c_i}\Vert$, the velocity direction of the agent itself ${\hat{\mathbf {v}}}_i = \mathbf {v}_i / \Vert \mathbf {v}_i\Vert$, the agent force (its action) $\mathbf {F}_i$ and the direction of the entire group velocity ${\hat{\mathbf {v}}}_{gc} = \mathbf {v}_{gc} / \Vert \mathbf {v}_{gc} \Vert$ for $\mathbf {v}_{gc} = \frac{1}{N} \sum \limits _{i = 1}^{N} \mathbf {v}_i$.

The emergent swirling motion is quantified by the average angular velocity of the swarm, which is defined as

$$\begin{aligned} {\varvec{\Omega }} = \frac{1}{N}\sum _{i = 1}^N \left( \mathbf {v}_i - \mathbf {v}_{gc}\right) \times \frac{\mathbf {x}_i - \mathbf{x}_{gc}}{\Vert \mathbf {x}_i - \mathbf{x}_{gc}\Vert ^2}, \end{aligned}$$

where the radius vector $\mathbf{x}_{gc}$ and velocity $\mathbf {v}_{gc}$ of the group center (i.e. of the center of mass) have been given above.

**Figure 7:**
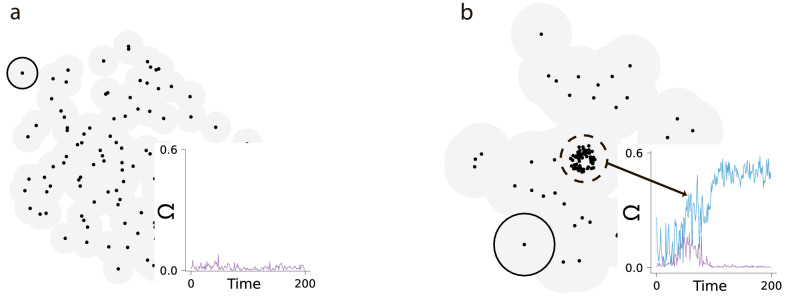
Typical swarm configurations for different size of the perception zone as compared to the size of the group. Configurations are shown for $100$ agents. Lite-gray shadows designate the perception zones of the animals (encircled for a single agent), while small black circles show the individual comfort zones. (**a**) When the size of the perception zone is significantly smaller than the group size a chaotic motion with zero angular velocity is observed. Inset: Time dependence of the average angular velocity $\Omega (t)$ of the swarm. (**b**) As the size of the perception zone increases, swirling emerges in a subgroup. In this case swirling coexists with the chaotic motion of the rest of the group. Inset: Time dependence of the average angular velocity $\Omega (t)$ of the whole group (purple) and of the swirling subgroup (blue).

Note that the emergence of swirling quantified by non-zero $\Omega$ requires the condition that all agents resides within the perception zone of each animal. If this condition is strongly violated the swirling is lacking, $\Omega =0$, see Fig. 7a. If the perception zone of the agents is smaller, but comparable with the dimension of the whole group, the swirling may arise in a subgroup, see Fig. 7b. In this case swirling coexists with a chaotic motion in the rest of the group.

### Physical background and algorithms for RL of efficient locomotion of a swarm

A system of spherical particles moving in viscous fluid at small Reynolds numbers may be described by the so-called Rodne-Prager theory^52^, which goes beyond the classical Oseen theory^53^. This theory may be formulated in the form of the velocity of $i$-th agent, $\mathbf{v}_i$, resulting from the forces $\mathbf{F}^e_j$, $j = 1, 2, \ldots , N$, applied to all $N$ agents by the environment:

2 $$\begin{aligned} \mathbf{v}_i = -\sum _{j=1}^N {\hat{\zeta }}_{ij} \mathbf{F}^e_j \end{aligned}$$

with the matrix of friction coefficients,

3 $$\begin{aligned} {\hat{\zeta }}_{ij} =\frac{1}{6 \pi \eta a} \left[ \frac{3a}{4 x_{ij}} \left( {\hat{I}} + {{ \bar{\mathbf{x}}}}_{ij}{{ \bar{\mathbf{x}}}}_{ij}^{\mathrm {T}}\right) + \frac{1}{2} \left( \frac{a}{x_{ij}} \right) ^3 \left( {\hat{I}} -3 {{ \bar{\mathbf{x}}}}_{ij}{{ \bar{\mathbf{x}}}}_{ij}^{\mathrm {T}} \right) \right] ; \qquad {\hat{\zeta }}_{ii} = \frac{1}{6\pi \eta a} {\hat{I}}, \end{aligned}$$

where ${\hat{I}}$ is a unit matrix, $a$ is the agent radius, $\eta$ is the fluid viscosity and $\mathbf{x}_{ij} = \mathbf{x}_{i}-\mathbf{x}_{j}$ is the radius vector joining two agents. Finally, ${{ \bar{\mathbf{x}}}}_{ij} = \mathbf{x}_{ij}/\Vert \mathbf{x}_{ij}\Vert$ is the unit inter-agent vector.

We assume that the motion is always over-damped, so that the velocity of an agent immediately relaxes to a uniform, time-independent value, corresponding to the set of forces $\mathbf{F}^e_i$, as in Eqs. (2) and (3), see SM. Then the power dissipated by $i$-th agent may be written as $\left( \mathbf{v}_i - \mathbf{v}_{f,i} \right) \cdot \mathbf{F}^a_{i}$, where $\mathbf{F}^a_i$ is the force exerted by $i$-th agent on the medium and $\mathbf{v}_{f,i}$ is the fluid velocity at the location of $i$-th agent. By the third Neuton’s law the force exerted by a uniformly moving body on a medium equals to the force acting on the body back from the medium. Hence the total power dissipated by a swarm of $N$ agents reads,

4 $$\begin{aligned} P =\sum _{i=1}^N P_i = \sum _{i=1}^N \left( \mathbf{v}_i - \mathbf{v}_{f,i} \right) \cdot \mathbf{F}^a_{i} = (6 \pi \eta a)^{-1} \sum _{i=1}^N \left( {\mathbf {F}_i^a}\right) ^2. \end{aligned}$$

To obtain the last part of Eq. (4), we observe that the local fluid velocity follows from Eqs. (2) and (3): $\mathbf{v}_{f,i}=\mathbf{v}_i+ \zeta _{ii} \mathbf{F}^e_{i}$, so that $(\mathbf{v}_i - \mathbf{v}_{f,i})= -\mathbf{F}^e_{i}/(6 \pi \eta a) = \mathbf{F}^a_{i}/(6 \pi \eta a)$.

Consider a swarm where all animals move with the same absolute velocity in the same direction. Then, as it follows from Eqs. (2), (3) and (4), the total dissipation $P$ will be determined by the set of vectors $\mathbf{x}_{ij}$, $i, j = 1, \ldots , n$, that define the mutual disposition of the agents. To find the optimal swarm configuration, we need to minimize $P$. This is a well-posed optimization problem with a straightforward solution; the result is given in Fig. 4. Interestingly, the optimal configuration for a pack of three animals corresponds to a triangle with a base along the direction of motion, that is, two equivalent configurations are possible, see Fig. 4.

Our next goal is to check whether the RL is able to find the optimal configurations which are known. To solve this problem we consider a group of animals comprising of a leader moving in fixed direction with a constant velocity and several followers which aim to retain their comfort zone w.r.t other agents and the leader; simultaneously they strive to minimize the dissipation of energy. The agents (followers) are aware of the following information:

1. Whether the closest agent breaks into the comfort zone $d_{cz}$:
  $$\begin{aligned} S_{cz}^i = H\bigg (d_{cz} - \Vert \mathbf {x}_{i} - \mathbf {x}_{c_i}\Vert \bigg ), \qquad c_i = \arg \min \limits _{j \ne i} \Vert \mathbf {x}_{i} - \mathbf {x}_{j}\Vert , \end{aligned}$$
  where $\mathbf {x}_i$ is the $i$-th agent position and $H(x)$ is the Heaviside step function.
2. Whether the agent approaches the closest agent:
  $$\begin{aligned} S_{ca}^i = H\left( -\frac{d}{dt} \Vert \mathbf {x}_i - \mathbf {x}_{c_i}\Vert \right) . \end{aligned}$$
3. Whether the agent resides within a target distance from the leader:
  $$\begin{aligned} S_d^i = H\bigg ( d_t - \Vert \mathbf {x}_i - \mathbf {x}_l\Vert \bigg ), \end{aligned}$$
  where $\mathbf {x}_l$ is the leader position and $d_t$ specifies the range of target distances to the leader.

We defined a reward, as explained in the Algorithm 3. The most desirable for the agent is to reside within the target distance to the leader. However, the agent is penalized if someone brakes into its comfort zone. If an agent is not within the target distance to the leader, we reward a pursuit of the leader. If an agent resides within the target distance to the leader, and there are no neighbours in the agent’s comfort zone, it is penalized for the energy consumption. In our simulations the value of $d_t$ was large enough to accommodate the whole swarm with an optimal configuration.

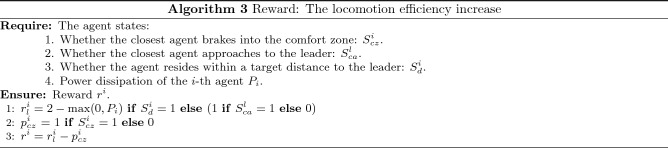



In this scenario, each agent is also aware about its velocity w.r.t. the “laboratory” system, that is, w.r.t. fluid at rest $\mathbf {v}_i$, the relative agents’ velocities: $\mathbf {v}_{ij} = \mathbf {v}_{j} - \mathbf {v}_{i}$, the agent force (its action) $\mathbf {F}^a_i$ and the relative agents positions.

## Supplementary information

Supplementary Information.
