# Supplementary material for: Why animals swirl and how they group
Source: Sci Rep. 2021 Oct 21;11:20843. doi: 10.1038/s41598-021-99982-7 (PMC8531037; doi:10.1038/s41598-021-99982-7)
Supplement: Supplementary file 1 — Supplementary Information. [file 41598_2021_99982_MOESM1_ESM.pdf]

Supplementary Material for
Why do animals swirl and how do they group?

Egor E. Nuzhin[1], Maxim E. Panov[1] and Nikolai V. Brilliantov[1] ✉
[1] *Skolkovo Institute of Science and Technology, 121205, Moscow, Russia.* ✉*e-mail: nb144@leicester.ac.uk*

PACS numbers:

A. Details of the neural networks and learning algorithms

Let's denote by $r(\mathbf{s}_t)$ a reward function, that depends on the agent state $\mathbf{s}_t$ at time t. The overall goal of an agent immersed in an environment is to achieve the largest return during its life from time $k = t$ till $k = t + T$:

$$R_t = \sum_{k=0}^{T} \gamma^k r(\mathbf{s}_{k+t}), \qquad \text{where } \gamma \in [0, 1).$$

The environment is usually random and distributed according to the distribution $P(\mathbf{s}_{t+1} \mid \mathbf{s}_t, \mathbf{a}_t)$ which defines a probability that the next state is $\mathbf{s}_{t+1}$, provided that being in a previous state $\mathbf{s}_t$ an agent used the action $\mathbf{a}_t$. The action policy is also given by the probability density function $\pi_\theta(\mathbf{a} \mid \mathbf{s})$. It defines the probability of an agent to use an action $\mathbf{a}$ if the agent current state is $\mathbf{s}$. The parameter θ is the subject of learning, that defines the specific action policy. The policy is trained to maximize the average expected reward $J[\pi_\theta]$ for the whole time range from $k = t$ to $k = t + T$:

$$J[\pi_\theta] = \mathbb{E}_{\Pi_\theta(A|S)P(S,A)}\left[R_t\right].$$

Here $A = \{\mathbf{a}_t, \dots, \mathbf{a}_{t+T}\}$ are the agent actions, $S = \{\mathbf{s}_t, \dots, \mathbf{s}_{t+T+1}\}$ are the agent states and

$$\Pi_\theta(A \mid S) = \prod_{k=0}^{T} \pi_\theta(\mathbf{a}_{t+k} \mid \mathbf{s}_{t+k}) \quad \text{and} \quad P_\theta(S, A) = \prod_{k=0}^{T} \pi_\theta(\mathbf{s}_{t+k+1} \mid \mathbf{s}_{t+k}, \mathbf{a}_{t+k}).$$

Usually, the maximization of the expected reward is analytically impossible. Moreover, the integration over the policy and the environment probability density functions cannot be computed exactly. The numerical integration over an episode (all time steps) is computationally challenging. Here we apply Monte-Carlo gradient estimate [4], as one of the approaches to overcome this difficulty.

Consider the equality:

$$\nabla_\theta J[\pi_\theta] = \mathbb{E}_{\Pi_\theta(A|S)P_\theta(S,A)}\left[R_t \sum_{k=0}^{T} \nabla_\theta \log\bigl(\pi_\theta(\mathbf{a}_{t+k} \mid \mathbf{s}_{t+k})\bigr)\right].$$

If we know the complete history from the state-action distribution, then we can find an unbiased estimate of the policy gradient:

$$\nabla_\theta \tilde{J}[\pi_\theta] = \tilde{R}_t \sum_{k=0}^{T} \nabla_\theta \log\bigl(\pi_\theta(\tilde{\mathbf{a}}_{t+k} \mid \tilde{\mathbf{s}}_{t+k})\bigr),$$

where $\tilde{R}_t$ is the return received for the entire episode of simulation, $\tilde{\mathbf{s}}_t$ is an agent state at time t and $\tilde{\mathbf{a}}$ is an agent action at time t. Then, using gradient descent, episode after episode, we will approach the optimal policy.

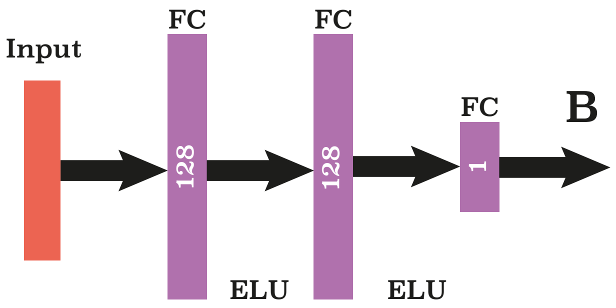

FIG. 1: The architecture of the neural network for the baseline network.

1. *Baseline*

There is an important property of the Monte-Carlo policy gradient estimate. We can supplement this function with an arbitrary shift called baseline value [3] $B(s_t, a_t)$ and this shift does not change the expectation:

$$\nabla_\theta J[\pi_\theta] = \mathbb{E}_{\Pi_\theta(A|S)P_\theta(S,A)}\left[\sum_{k=0}^{T}\left(R_{t+k} - B(\mathbf{s}_{t+k})\right)\nabla_\theta \log\bigl(\pi_\theta(\mathbf{a}_{t+k} \mid \mathbf{s}_{t+k})\bigr)\right].$$

We may require this function to approximate the expected return, and then we will find that the variance of the gradient estimate decreases. In practice, we train the baseline as a neural network to predict the expected reward, based on the return of the episode and the mean squared error loss. Hence the resulting Monte-Carlo gradient estimate with the reduced variance reads,

$$\nabla_\theta \hat{J}[\pi_\theta] = \sum_{k=0}^{T} \tilde{A}_{t+k}\nabla_\theta \log\bigl(\pi_\theta(\tilde{\mathbf{a}}_{t+k} \mid \tilde{\mathbf{s}}_{t+k})\bigr),$$

where $\tilde{A}_t = \tilde{R}_t - B(\tilde{\mathbf{s}}_t)$ is an estimate of the advantage function. The architecture of the baseline neural network (NN) is depicted in Fig. 1.

As a starting point one need to initialize the parameters of a NN. Usually, the parameters are set up randomly. Here we use a uniform parameter distribution:

$$\mathbf{W_k}, \mathbf{b_k} \sim \mathcal{U}\left(-\frac{1}{\sqrt{f_{in}^k}}, \frac{1}{\sqrt{f_{in}^k}}\right),$$

where $\mathcal{U}(x, y)$ is a uniform function defined on the interval (x, y) and f_{in}^k is a size of k-th layer input. It corresponds to $f_{in}^1 = 8$ for the first layer and $f_{in}^2 = f_{in}^3 = 128$ for the second and third layers.

The learning was performed by Adam optimizer [1]. The step size parameter was chosen $\alpha = 0.00001$, exponential decay rates $\beta_1 = 0.9, \beta_2 = 0.999$.

The discount factor was specified to $\gamma = 0.9$. The length of the episode was 1000 steps.

2. *Normalized Advantage*

We also use the normalized advantage for the stability purposes [5]. The normalization in our work, means, that we subtract the mean from the values of the advantage history and divide them by the standard deviation:

$$\mu_A = \frac{1}{T+1}\sum_{k=0}^{T}\tilde{A}_{t+k}, \qquad \sigma_A^2 = \frac{1}{T+1}\sum_{k=0}^{T}\tilde{A}_{t+k}^2 - \mu_A^2.$$

That is, the normalized Monte-Carlo estimate of the policy gradient may be written as:

$$\nabla_\theta \hat{J}[\pi_\theta] = \sum_{k=0}^{T}\frac{\tilde{A}_{t+k} - \mu_A}{\sigma_A}\nabla_\theta \log\bigl(\pi_\theta(\tilde{\mathbf{a}}_{t+k} \mid \tilde{\mathbf{s}}_{t+k})\bigr).$$

3. Multi-agent Reinforcement Learning

There are many ways to model multi-agent systems [6]. We assume that each agent has the same policy and cannot transmit any information to any other agent. Thus, the policy depends only on a single agent state, and training n agents simultaneously allow us to obtain n gradient estimates per an episode. Two cases are possible: cooperative agents and competitive agents.

For the former case we assume, that all agents have the same reward, hence Monte-Carlo gradient estimate reads,

$$\nabla_\theta \hat{J}[\pi_\theta] = \sum_{i=1}^{N} \sum_{k=0}^{T} \left(\frac{\tilde{A}^i_{t+k} - \mu_A}{\sigma_A} \right) \nabla_\theta \log\bigl(\pi_\theta(\tilde{\mathbf{a}}^i_{t+k} \mid \tilde{\mathbf{s}}^i_{t+k})\bigr),$$

where

$$\tilde{A}^i_t = \tilde{R}_{t+k} - B(\tilde{\mathbf{s}}^i_{t+k})$$

and $\tilde{\mathbf{s}}^i_t$ is the state of the i-th agent at time t, $\tilde{\mathbf{a}}^i_t$ is the action of the n-th agent at time t and $\tilde{R}_t$ is the return at time t based on the states of all agents.

For the latter case we use different rewards for each agent. Thus, Monte-Carlo gradient estimate reads,

$$\nabla_\theta \hat{J}[\pi_\theta] = \sum_{i=1}^{N} \sum_{k=0}^{T} \left(\frac{\tilde{A}^i_{t+k} - \mu_A}{\sigma_A} \right) \nabla_\theta \log\bigl(\pi_\theta(\tilde{\mathbf{a}}^i_{t+k} \mid \tilde{\mathbf{s}}^i_{t+k})\bigr),$$

where

$$\tilde{A}^i_t = \tilde{R}^i_t - B(\tilde{\mathbf{s}}^i_t)$$

and $\tilde{R}^i_t$ is the return at time t of the i-th agent based on its states.

4. Details of the escort scenario B

Let $a_{\max}$ be a maximal possible acceleration. We implement the agent policy as follows: if a speed chosen by an agent is feasible, i.e., it results in the acceleration smaller than $a_{\max}$, the agent follows the same actions as in the previous model. In the opposite case it chooses to move in the same direction with the acceleration, equal to $a_{\max}$. If both actions are not possible (see below), the agent slows down keeping its direction of motion.

Now we explain this in more detail: If the desired agent velocity results in a greater acceleration then $a_{\max}$, then the agent speed is taken to be $c\frac{\mathbf{v}_d}{\|\mathbf{v}_d\|}$ where $0 \le c \le 1$. Here c is a pure number which is the solution of the following equation:

$$\|\mathbf{v}_{-1} - \mathbf{v}_n c\| = a_{\max} \cdot dt,$$

where $\mathbf{v}_{-1}$ is the velocity at the last time step and $\mathbf{v}_n = \frac{\mathbf{v}_d}{\|\mathbf{v}_d\|}$ is the desired direction at the next time step. Squaring the equation, we get:

$$\|\mathbf{v}_n\|^2 c^2 - 2\mathbf{v}_{-1}^{\mathrm{T}} \mathbf{v}_n c + \|\mathbf{v}_{-1}\|^2 - (a_{\max} \cdot dt)^2 = 0$$

and

$$c = \frac{\mathbf{v}_{-1}^{\mathrm{T}} \mathbf{v}_n \pm \sqrt{(\mathbf{v}_{-1}^{\mathrm{T}} \mathbf{v}_n)^2 - \|\mathbf{v}_n\|^2(\|\mathbf{v}_{-1}\|^2 - (a_{\max} \cdot dt)^2)}}{\|\mathbf{v}_n\|^2}.$$

If c does not exist in the range $0 \le c \le 1$, then the agent slows down. In this case, we define the agent velocity as $v = \mathbf{v}_{-1} k$ where $0 \le 1$. We define k as the solution of the following equation:

$$\|\mathbf{v}_{-1} - \mathbf{v}_{-1} k\| = a_{\max} \cdot dt.$$

Similarly, we obtain:

$$k = 1 - \frac{a_{\max} \cdot dt}{\|\mathbf{v}_{-1}\|}.$$

This policy is presented in the Algorithm 1.

Algorithm 1 The agent policy for the Escort Problem with limited acceleration

---

Require:
The agent's desired velocity $\mathbf{v}_d$ chosen with a policy;
The velocity at the last time step $\mathbf{v}_{-1}$;
The maximum acceleration $a_{\max}$;
The time discretization step dt.
Ensure: The agent velocity at the next time step $\mathbf{v}$.

```
if ‖v_d − v_{−1}‖ / dt ≤ a_max then
    v = v_d
else
    v_n = v_d / ‖v_d‖
    c_1 = (v_{−1}^T v_n + sqrt((v_{−1}^T v_n)^2 − ‖v_n‖^2 (‖v_{−1}‖^2 − (a_max·dt)^2))) / ‖v_n‖^2 ,  c_2 = (v_{−1}^T v_n − sqrt((v_{−1}^T v_n)^2 − ‖v_n‖^2 (‖v_{−1}‖^2 − (a_max·dt)^2))) / ‖v_n‖^2
    if (0 ≤ c_1 ≤ 1) | (0 ≤ c_2 ≤ 1) then
        if (0 ≤ c_1 ≤ 1) & (0 ≤ c_2 ≤ 1) then
            c = max(c_1, c_2)
        else
            c = c_1 if 0 ≤ c_1 ≤ 1 else c_2
            v = c · v_n
        end if
    else
        k = 1 − (a_max·dt) / ‖v_{−1}‖
        v = max(0, k) v_{−1}
    end if
end if
```

---

5. *Smooth reward functions*

As it has been already stated in the main text, the jerkily motion with non-smooth trajectories in the escort scenario A is not related to the discontinuity of the step-function, involved in the estimate of the reward. To prove this we consider the respective smooth function (see the Algorithm 2). The Boolean logic applied in the discontinues scenario was substituted by fuzzy logic. In this approach the agent states remain the same, but the reward function smoothly varies, depending on the distance to the target and the rate of the distance change.

The results depicted in Fig. 2 prove the concept that the jerkily trajectories originate exclusively due to the physical nature of the according strategy and not due to mathematical artefacts.

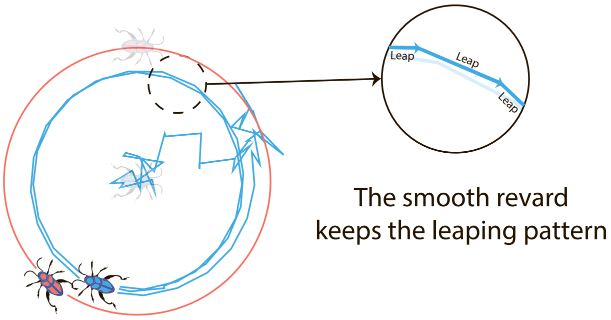

FIG. 2: The example of the jerkily motion with non-smooth trajectories in the escort scenario A for the case of smooth reward function. This proves the physical nature of the jerkily strategy.

Algorithm 2 Smooth Reward – Escort problem

Require: Environment parameters:
1. Accuracy: e_t.

Require: Environment states:
1. Follower's distance to the target: s.
2. Follower's speed to the target: $v = -\frac{d}{dt}s$.

Ensure: Reward r.

1: $\sigma(x) = \min(\max(x + \frac{1}{2}, 0), 1)$
2: $S_d = \sigma(\frac{e_t - s}{e_t})$ (fuzzy representation of: whether the follower resides within a range of target distances from the leader)
3: $S_a = \sigma(\frac{v}{e_t})$ (fuzzy representation of: whether the follower approaches the leader)
4: $r = -1 + \max(S_a, S_d) + S_d$ (The agent's confidence (fuzzy membership) weighed reward. Smooth representation of the discrete reward: **if** $S_d = 1$ **else** (0 **if** $S_a = 1$ **else** -1))

B. Swirling Motion

The emergence of swirling may be quantified by the average angular velocity Ω. It is defined in Sec. III of the main text. The time evolution of this quantity is depicted in the Inset in Fig. 3a of the main text. Here, in Fig. 3 we present a full-scale plot for the evolution of Ω, along with the according swirling patterns at different self-organization stages.

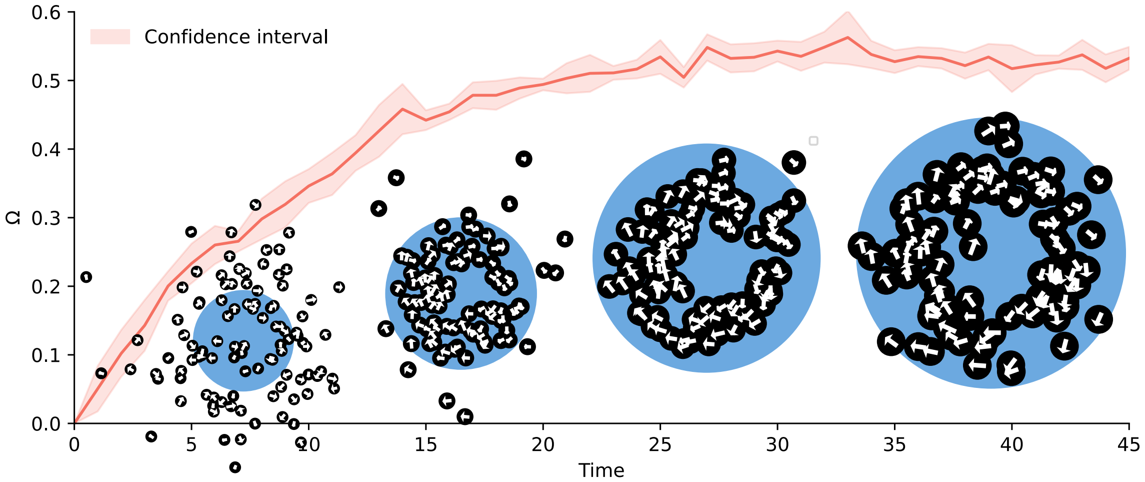

FIG. 3: The time evolution of the average angular velocity of a swarm Ω, as defined in Sec. III of the main text. Under the curve the successive swirling patterns corresponding to the different stages of self-organization are shown.

C. Details of the training of swirling with the external force

We assume that the swirling motion helps a group of animals to resist external perturbations in the environment. To check this assumption we have incorporated an additional external force in the environment and measured the group center shift in the external force direction. Each episode, we changed the direction of the external force with a uniform angle distribution. During the simulations, we randomly modulated the force strength using the stretched exponential distribution. The experiment repeated ten times for each average force. The comparison of the center shift (in the direction of the acting force) for swirling and non-swirling swarms, Fig. 4, shows that the trained to swirl swarm resist the external force up to 100 times more efficiently than the untrained one. Hence we conclude that orbiting the group center results in extremely high resistance to external perturbations. Although very simple, the strategy can help the agents (animals) to cope with the hostile environment. Hence the stunning swirling motion is not a random arrangement or a behavior error. We see that this behavior plays a crucial role in their survival. Of course, complex organisms can learn more efficient strategies, but this strategy to resist external influences may be the most desirable for simple bests.

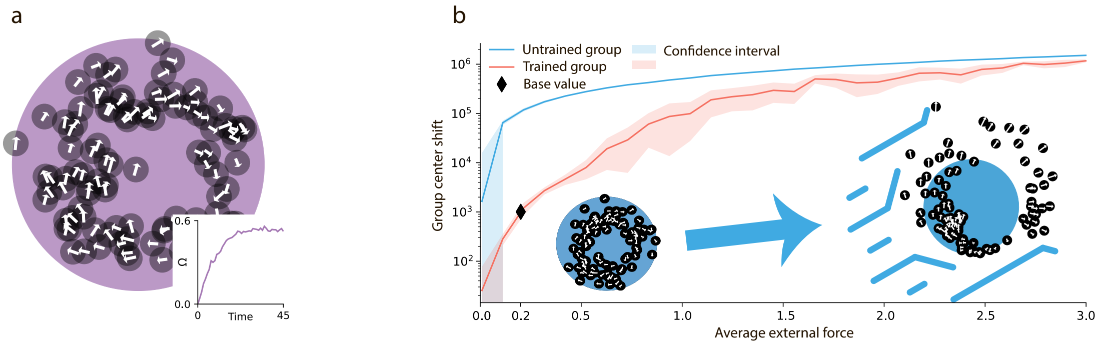

FIG. 4: The comparison of an ability to resist the external perturbation of an untrained and trained swarms.

The effect of the resistance of a trained swarm to external perturbations is very robust. We tested the resistance stability for different agents' comfort zone (w.r.t. the group center), number of agents in a swarm, and types of random force acting on agents. For the general stretched exponential distribution of the random force

$$P(f \mid f_0, \beta) = \frac{1}{f_0} \frac{\beta}{\Gamma(1/\beta)} \cdot e^{-(f/f_0)^{\beta}},$$

we vary the stretching exponent β keeping the mean force fixed. The according scale factor f_0 of the distribution with the fixed mean μ and the exponent β reads,

$$f_0 = \frac{\Gamma(1/\beta)}{\Gamma(2/\beta)} \cdot \mu.$$

Our simulations confirm that the ability of a swarm to resist the external perturbations does not qualitatively change when the parameters of the swarm or the perturbation parameters vary. This is illustrated in Fig. 5.

We also analyze the probability to find an agent at some position w.r.t. the group center (see Fig. 6). The first model, see Fig. 6a, was trained without an external force, while the second one, see Fig. 6b – with an additional perturbation. Fig. 6 illustrates an interesting result: When the external force was lacking, the agents moved inside a ring only; under the influence of the external force the group center became occupied.

The swirling model was trained by a two-phase procedure. First, we trained the policy in the environment with only two agents in the group. After the policy convergence, we retrained the policy in the environment with 100 agents as was described earlier.

D. Details of policy implementation: Bounded normal distribution

In our study we consider different types of action, which could be a variation of an agent force or speed. Let **a** be real valued n-dimensional vector (a two dimensional continuous variable in our case). To model the action policy we use the parameterized continuous probability distribution [4]. During a learning process a policy usually converges to a definite one with a minimal uncertainty. Thus, the type of the distribution has a minor impact on the learning result. For simplicity we choose a normal distribution,

$$\mathcal{N}\big(\mathbf{x} \mid \boldsymbol{\mu}(\mathbf{s}), diag(\boldsymbol{\sigma}(\mathbf{s})^2)\big),$$

where $\boldsymbol{\mu}(\mathbf{s})$ is the mean value vector of the normal distribution and $\boldsymbol{\sigma}(\mathbf{s})$ is the vector of the variances; **s** denotes the state of the system. The functions, $\boldsymbol{\mu}(\mathbf{s})$ and $\boldsymbol{\sigma}(\mathbf{s})$, are to be specified by the neural network in the course of learning.

The standard normal distribution does not comply with physical constraints of our system (e.g. the velocity of an agent cannot be too high). Hence we need to modify the normal distribution accordingly – it should have only bounded random variables. We call such a distribution "a bounded normal distribution". We assume that the norm of a variable **a**, specifying an action is bounded by some quantity $a_{\max}$. Then we define a bounded distribution through n-dimensional vector-function $\mathbf{a}(\mathbf{x})$ of the normal random variables $\mathbf{x}$, limited to a n-dimensional sphere of radius

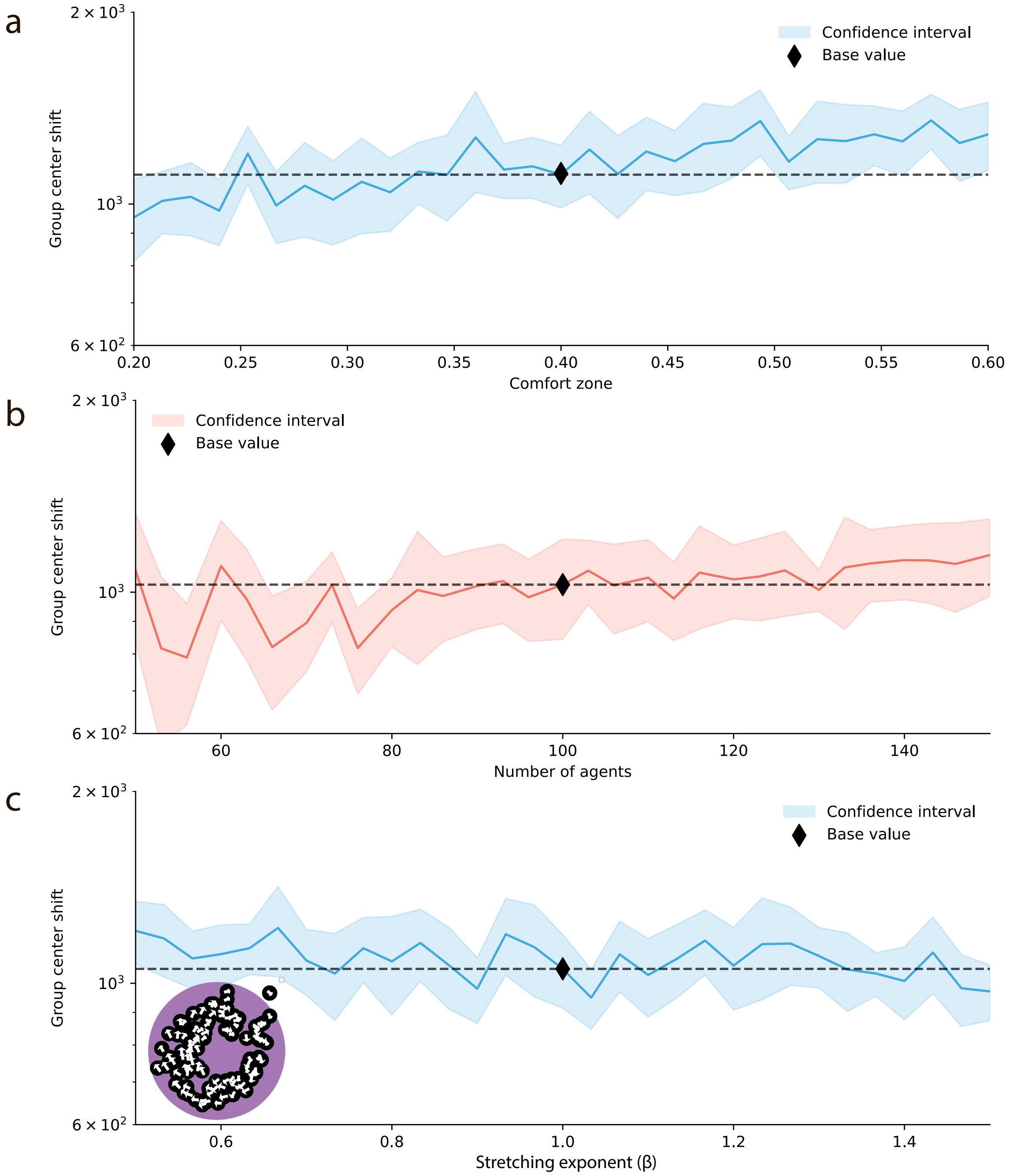

FIG. 5: The dependence of the shift of a trained group center on the swarm properties and the perturbation parameters. (a) The dependence of the shift on the size of the comfort zone of the swarm. (b) The dependence of the shift on the number of agent in the swarm. (c) The dependence of the shift on the parameter (β) of the stretched exponential distribution with fixed mean force. The marker indicates the value of the parameters used in the main text.

$a_{\max}$:

$$\mathbf{a}(\mathbf{x}) = a_{\max}\frac{\mathbf{x}}{\|\mathbf{x}\|}e^{-\|\mathbf{x}\|^2}.$$

Then, the probability density for the n-dimensional vector $\mathbf{a}(\mathbf{x})$ can be written as follows:

$$\mathcal{BN}^n_{a_{\max}}(\mathbf{a}(\mathbf{x}) \mid \mathbf{s}) = \mathcal{N}\big(\mathbf{x} \mid \boldsymbol{\mu}(\mathbf{s}), diag(\boldsymbol{\sigma}(\mathbf{s})^2)\big)\, J^{-1}_{\mathbf{a}(\mathbf{x})},$$

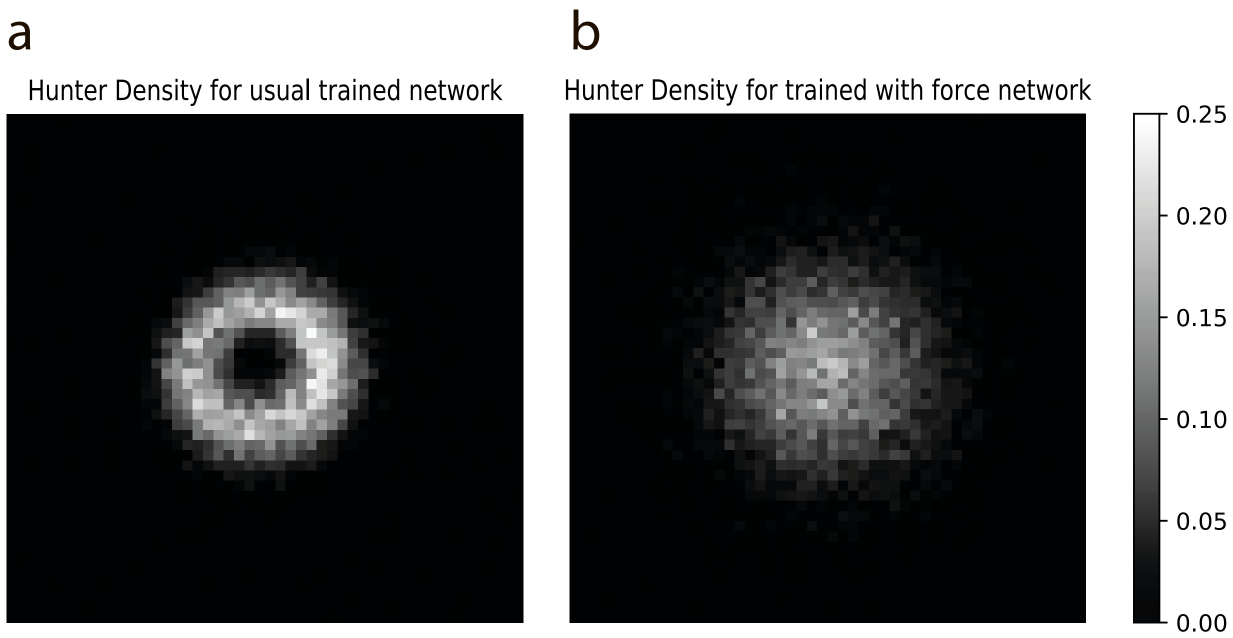

FIG. 6: The probability density to find an agent at a given position w.r.t the group center for (a) a trained swarm without an external force and (b) a trained swarm with the external perturbation.

where $J_{\mathbf{a}(\mathbf{x})}$ is the Jacobian of $\mathbf{a}(\mathbf{x})$:

$$J_{\mathbf{a}(\mathbf{x})} = \det\big(\nabla_{\mathbf{x}}\mathbf{a}(\mathbf{x})\big).$$

Let us find the Jacobi matrix. The following equality holds true for an arbitrary vector function $\mathbf{f}(\mathbf{x})$ and an arbitrary scalar function $g(\mathbf{x})$:

$$\nabla_{\mathbf{x}}\left[\mathbf{f}(\mathbf{x})g(\mathbf{x})\right] = \frac{d\mathbf{f}(\mathbf{x})}{d\mathbf{x}}g(\mathbf{x}) + \frac{dg(\mathbf{x})}{d\mathbf{x}}\mathbf{f}(\mathbf{x})^{\mathrm{T}}.$$

Then, applying the above equation, the Jacobi matrix may be found:

$$\nabla_{\mathbf{x}}\mathbf{a}(\mathbf{x}) = a_{\max}\left(\frac{e^{-\|\mathbf{x}\|^2}}{\|\mathbf{x}\|}I + \nabla_{\mathbf{x}}\left[\frac{e^{-\|\mathbf{x}\|^2}}{\|\mathbf{x}\|}\right]\mathbf{x}^{\mathrm{T}}\right),$$

where

$$\nabla_{\mathbf{x}}\left[\frac{e^{-\|\mathbf{x}\|^2}}{\|\mathbf{x}\|}\right] = \nabla_{\|\mathbf{x}\|}\left[\frac{e^{-\|\mathbf{x}\|^2}}{\|\mathbf{x}\|}\right]\nabla_{\mathbf{x}}\|\mathbf{x}\|; \quad \nabla_{\mathbf{x}}\|\mathbf{x}\| = \frac{\mathbf{x}}{\|\mathbf{x}\|}; \quad \nabla_{\|\mathbf{x}\|}\left[\frac{e^{-\|\mathbf{x}\|^2}}{\|\mathbf{x}\|}\right] = -\left[\frac{1}{\|\mathbf{x}\|^2} + 2\right]e^{-\|\mathbf{x}\|^2}.$$

After a simple algebra we finally arrive at:

$$\nabla_{\mathbf{x}}\mathbf{a}(\mathbf{x}) = a_{\max}\frac{e^{-\|\mathbf{x}\|^2}}{\|\mathbf{x}\|}\left(I - \left[\frac{1}{\|\mathbf{x}\|^2} + 2\right]\mathbf{x}\mathbf{x}^{\mathrm{T}}\right).$$

The determinant of the Jacobi matrix (Jacobian of $\mathbf{a}(\mathbf{x})$) may be computed with the use of Weinstein-Aronszajn identity [2].

$$\mathcal{J}_{\mathbf{a}(\mathbf{x})} = -\left(a_{\max}\frac{e^{-\|\mathbf{x}\|^2}}{\|\mathbf{x}\|}\right)^n\left(1 - \left[\frac{1}{\|\mathbf{x}\|^2} + 2\right]\|\mathbf{x}\|^2\right) = 2\left(a_{\max}\frac{e^{-\|\mathbf{x}\|^2}}{\|\mathbf{x}\|}\right)^n\|\mathbf{x}\|^2.$$

Hence the final form for the probability density of $\mathbf{a}(\mathbf{x})$ reads:

$$\mathcal{BN}^n_{a_{\max}}(\mathbf{a}(\mathbf{x}) \mid \mathbf{s}) = \frac{1}{2\|\mathbf{x}\|^2}\left(\frac{\|\mathbf{x}\|e^{\|\mathbf{x}\|^2}}{a_{\max}}\right)^n \mathcal{N}\big(\mathbf{x} \mid \boldsymbol{\mu}(\mathbf{s}), diag(\boldsymbol{\sigma}(\mathbf{s})^2)\big).$$

For the two-dimensional problem, addressed here the last equation takes the form:

$$\mathcal{BN}^2_{a_{\max}}(\mathbf{a}(\mathbf{x}) \mid \mathbf{s}) = \frac{e^{2\|\mathbf{x}\|^2}}{2a^2_{\max}}\mathcal{N}\big(\mathbf{x} \mid \boldsymbol{\mu}(\mathbf{s}), diag(\boldsymbol{\sigma}(\mathbf{s})^2)\big).$$

E. The effective locomotion of two agents

Let us compare the energy dissipation of a solely moving alone, with that of paired agents. Suppose both agents move aligned, one after another, with a same constant velocity.

According to the discussion in the main text, the force, acting on a single agent reads

$$\mathbf{F}_{e_1} = -6\pi\eta_s a\mathbf{v}.$$

Since both agents move with a constant velocity, the total force acting on them equals zero. That is (see the main text for the notations),

$$\mathbf{F}_{a_i} = -\mathbf{F}_{e_i}.$$

Then, according to Eq.(3) of the main text, the power dissipation of a solely moving agent reads,

$$P_1 = \frac{\|\mathbf{F}_e\|^2}{6\pi\eta_s a} = 6\pi\eta_s a\|\mathbf{v}\|^2.$$

Since the agent velocities are the same, the force acting on them, may be found from the following equations: (we write it in a block form):

$$\begin{pmatrix}\mathbf{v}\\ \mathbf{v}\end{pmatrix} = -\begin{pmatrix}\zeta_{11} & \zeta_{12}\\ \zeta_{12} & \zeta_{11}\end{pmatrix}\begin{pmatrix}\mathbf{F}_{e_1}\\ \mathbf{F}_{e_2}\end{pmatrix},$$

where the Rodne-Prager approximation has been applied (see the main text). Due to the symmetry of the translation matrix and equal velocities, the agent forces are equal and read:

$$\begin{aligned}\mathbf{F}_e &= -\left(\zeta_{11} + \zeta_{12}\right)^{-1}\mathbf{v} \\ &= -6\pi\eta_s a\left(I + \frac{3}{4}\frac{a}{r}\left(I + \hat{R}\hat{R}^T\right) + \frac{1}{2}\left(\frac{a}{r}\right)^3\left(I - 3\hat{R}\hat{R}^T\right)\right)^{-1}\mathbf{v} \\ &= -6\pi\eta_s a\left[K_\perp\hat{R}_\perp\hat{R}_\perp^T - K_\parallel I\right]\mathbf{v},\end{aligned} \tag{1}$$

where $\hat{R}$ is the unit inter-agent vector, $r = \|R\|$ is the distance between the agents,

$$\hat{R}_\perp = \begin{pmatrix}0 & -1\\ 1 & 0\end{pmatrix}\hat{R}, \quad K_\perp = \frac{2}{2\left(\frac{a}{r}\right)^3 - 3\frac{a}{r} - 2} + \frac{4}{2\left(\frac{a}{r}\right)^3 + 3\frac{a}{r} + 4}$$

and

$$K_\parallel = \frac{2}{2\left(\frac{a}{r}\right)^3 - 3\frac{a}{r} - 2}.$$

If the agents move aligned, one after another, at a constant velocity, their radius-vectors are parallel to the velocity

$$\mathbf{v} = \|\mathbf{v}\|\hat{R}.$$

Then the force, acting on the agents takes the from:

$$\mathbf{F}_e = 6\pi\eta_s a\|\mathbf{v}\| \frac{2}{2\left(\frac{a}{r}\right)^3 - 3\frac{a}{r} - 2}.$$

Using again Eq.(3) from the main text we find the power dissipation for each for the agents:

$$P_2 = \frac{\|\mathbf{F}_e\|^2}{6\pi\eta_s a} = 6\pi\eta_s a\|\mathbf{v}\|^2 \frac{4\left(\frac{r}{a}\right)^6}{\left(\left(\frac{r}{a}\right)^2\left(2\frac{r}{a}+3\right)-2\right)^2}.$$

The ratio of the power dissipation of a paired agent and solely moving agent reads,

$$\frac{P_2}{P_1} = \frac{4\left(\frac{r}{a}\right)^6}{\left(\left(\frac{r}{a}\right)^2\left(2\frac{r}{a}+3\right)-2\right)^2}.$$

Since the distance between the agents is limited by their size, that is, $\frac{r}{a} \geq 2$, the ratio of power dissipation is less than one, $\frac{P_2}{P_1} \leq 1$. In other words, the dissipation of a solely moving agent is larger than that of a paired agent.

$$P_2 < P_1.$$

The dependence of the ratio of the power dissipation on the relative distance between the agents is shown in Fig. 7. The minimum power dissipation is archived when the agents move as close as possible, that is, at the distance of $2a$.

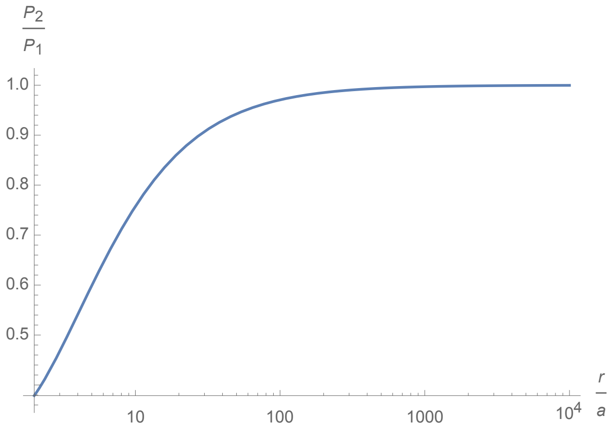

FIG. 7: The ratio of the power dissipation P_2 of a paired agent and of a solely moving agent, P_1, as a function of distance r between the paired agents. Note that only $r > 2a$, where a is the agent size (radius), correspond to possible distances.

Now we define cumulative loss function of the agents:

$$L = P_1 + P_2,$$

subject to $\|R\| \geq 2r_{cz}$, where P_1,P_2 are the dissipated powers, R is a vector distance between the agents, r_{cz} is a comfort zone.

The global optimum is possible when the derivative of the loss function is zero:

$$\frac{dL}{dR} = 0.$$

To find the optimum, we simplify the model using the assumption, that when the agents are in the optimal positions, they move uniformly without acceleration at a constant distance between them:

$$\mathbf{v}_{a_1} = \mathbf{v}_{a_2} = \mathbf{v} \equiv const; \quad R \equiv const.$$

If the agents move uniformly, the force of the environment equals to the opposite of the force exerted on the environment by the agent:

$$\mathbf{F}_{a_1} = -\mathbf{F}_{e_1}, \quad \mathbf{F}_{a_2} = -\mathbf{F}_{e_2}.$$

Moreover, the agents forces are equal, and according to Eq. (1) of the main text, read:

$$\mathbf{F}_{a_1} = \mathbf{F}_{a_2} = \mathbf{F}_e = -6\pi\eta_s a \left[K_\perp \hat{R}_\perp \hat{R}_\perp^T - K_\| I\right] \mathbf{v}.$$

Thus, the power dissipation depends on the environment force only (see Eq.(3) of the main text):

$$P_1 = P_2 = P = \frac{\|\mathbf{F}_e\|^2}{6\pi\eta_s a}.$$

It is convenient to write R and V in terms of unit vectors and their absolute values:

$$R = r\hat{R}, \quad V = v\hat{V},$$

where $\hat{R} = \frac{R}{\|R\|}$, $r = \|R\| \geq 0$, $\hat{V} = \frac{V}{\|V\|}$ and $v = \|V\| \geq 0$.

Then the environment force reads

$$\mathbf{F}_e = -6\pi\eta_s a v \left[K_\perp \hat{R}_\perp \hat{R}_\perp^T - K_\| I\right] \hat{V} = -6\pi\eta_s a v \left[K_\perp \hat{R}_\perp \cos\alpha - K_\| \hat{V}\right],$$

where α is an angle between V and $R_\perp$.

Then, the power dissipation may be written as a function of the angle β between R and V as:

$$P = 6\pi\eta_s a \left[\left(K_\perp^2 - 2K_\perp K_\|\right)\cos^2\alpha + K_\|^2\right] = 6\pi\eta_s a \left[\left(K_\perp^2 - 2K_\perp K_\|\right)\sin^2\beta + K_\|^2\right].$$

Since the distance between the agents is limited by their size, $\frac{r}{a} \geq 2$, and

$$K_\perp^2 - 2K_\perp K_\| \geq 0,$$

we conclude that the power dissipation is minimal when $\hat{v}||\hat{R}$ ($\beta = 0$), i.e. when the agents move aligned, one after another.

Thus, the optimal strategy of two agents is to move aligned, $\hat{v}||\hat{R}$, and as close as possible, $\|R\| = r_{cz}$.

We performed numerical experiments to confirm the optimal strategy of the agents in the fluid. We specified a unit agent radius, unit viscosity, and unit velocity. Besides, we limited the minimal speed of the agents as 0.65 m/sec. Without a speed limit the agents also achieve the optimum power dissipation moving one after another, as close as possible. The results are depicted in Fig. 8. Here we show the dissipated power as a function (encoded in color) of the inter-particle distance and angle between the inter-particle radius and direction of the velocity α. Fig. 8a illustrates the case when the speed limit is lacking, while in Fig. 8b the velocity constraint is applied. Finally, Fig. 8c demonstrates the surfaces of equal power dissipation.

F. Details of the RL for the effective locomotion of a group

The model was trained with a maximal force, equal to 0.5 N for 1000-seconds episode. The first experiment was conducted for a single agent-follower configuration. Fig. 9 shows that the algorithm converged to the same optimal configuration that was found before analytically and numerically. The optimal configuration is the aligned motion at a closest distance. This result proves that we can train a model to find complex optimal configurations with the help of RL.

Next, we trained the model for four agents, Fig. 10, and performed the numerical optimization to find the optimal configuration with a fixed velocity for the same parameters, Fig. 10. The result demonstrates that the RL can find the optimal disposition also for a multi-agent case.

Finally, we present Fig. 11, where the optimal configurations of the flocks up to 8 animals are shown. One can see complicated non-symmetric, w.r.t. the direction of motion, patterns for the case of the odd number of agents.

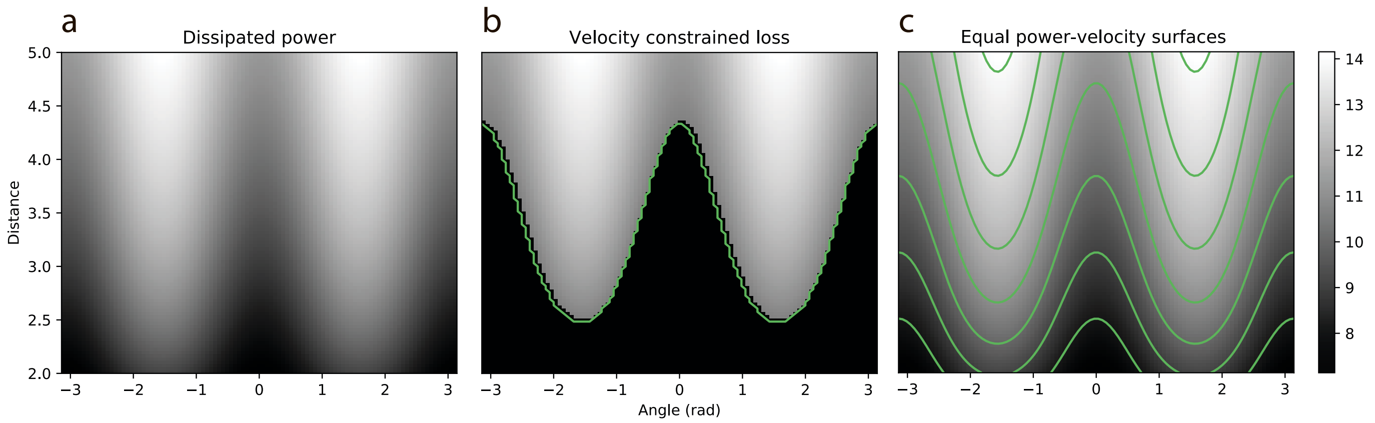

FIG. 8: The cumulative power dissipation of two agents moving at a constant speed as function of the inter-particle distance and angle α. (a) The power dissipation without speed constraints, (b) the power dissipation with a limitation of a minimal speed, (c) the power dissipation with the lines showing the surfaces of equal dissipation. The black region with zero loss corresponds to inaccessible speeds.

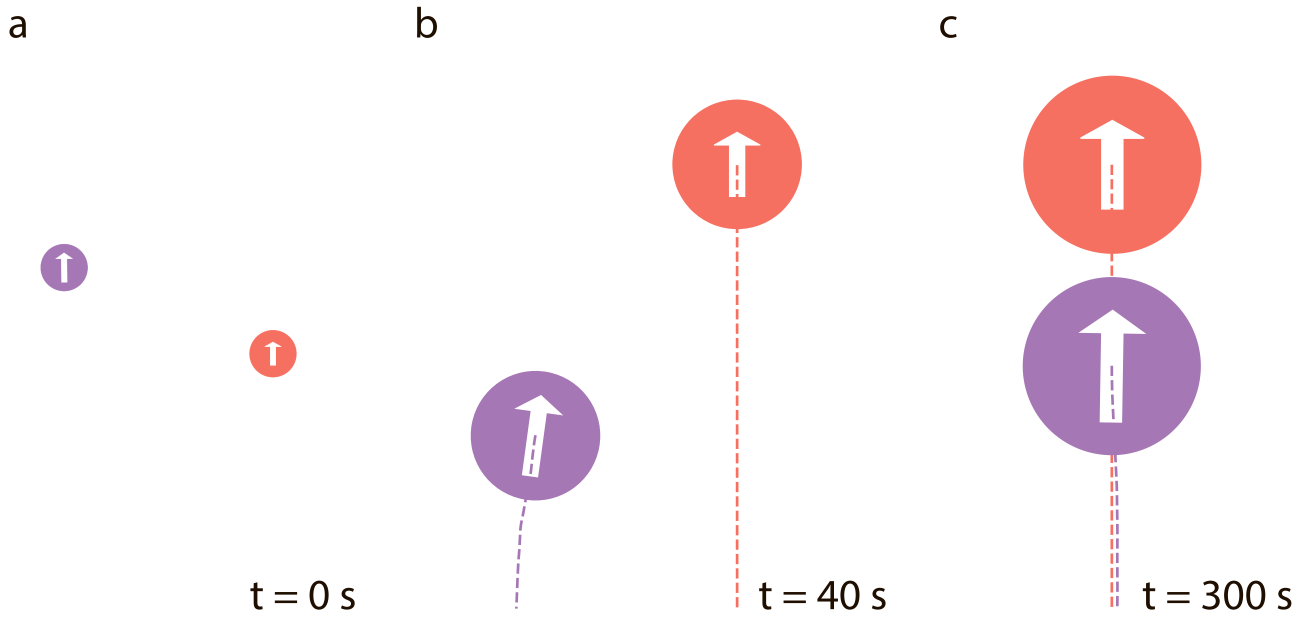

FIG. 9: Typical agent trajectories of the trained for efficient locomotion agents at time (a) t = 0 s, (b) t = 40 s and (c) t = 100 s. The violet circle is the agent-follower comfort zone, the red circle shows the leader comfort zone and the lines represent the agent trajectories. The (a), (b) and (c) panels correspond to the magnified view with the same size of the comfort zoen.

[1] D. P. Kingma and J. Ba. Adam: A method for stochastic optimization. *arXiv preprint arXiv:1412.6980*, 2014.
[2] C. Pozrikidis. *Introduction to Theoretical and Computational Fluid Dynamics 2nd Edition.* Oxford University Press; 2nd edition, 2011.
[3] J. Schulman, P. Moritz, S. Levine, M. Jordan, and P. Abbeel. High-dimensional continuous control using generalized advantage estimation. *arXiv preprint arXiv:1506.02438*, 2015.
[4] R. S. Sutton, D. A. McAllester, S. P. Singh, and Y. Mansour. Policy gradient methods for reinforcement learning with function approximation. In *Advances in neural information processing systems*, pages 1057–1063, 2000.
[5] H. P. van Hasselt, A. Guez, M. Hessel, V. Mnih, and D. Silver. Learning values across many orders of magnitude. In *Advances in Neural Information Processing Systems*, pages 4287–4295, 2016.
[6] K. Zhang, Z. Yang, and T. Başar. Multi-agent reinforcement learning: A selective overview of theories and algorithms. *arXiv preprint arXiv:1911.10635*, 2019.

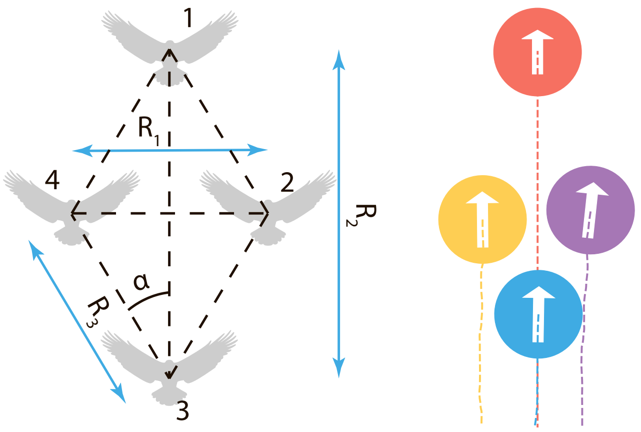

(a) Predicted configuration numerically

(b) Typical agent trajectories

FIG. 10: An example of the agents' displacement with a policy trained for locomotion efficiency increase. (a) The predicted configuration using numerical optimization with a constant velocity, (b) a typical configuration with the trained model's trajectories. The red circle describes the leader's comfort zone, and other circles show the agent-followers comfort zones. The lines represent trajectories.

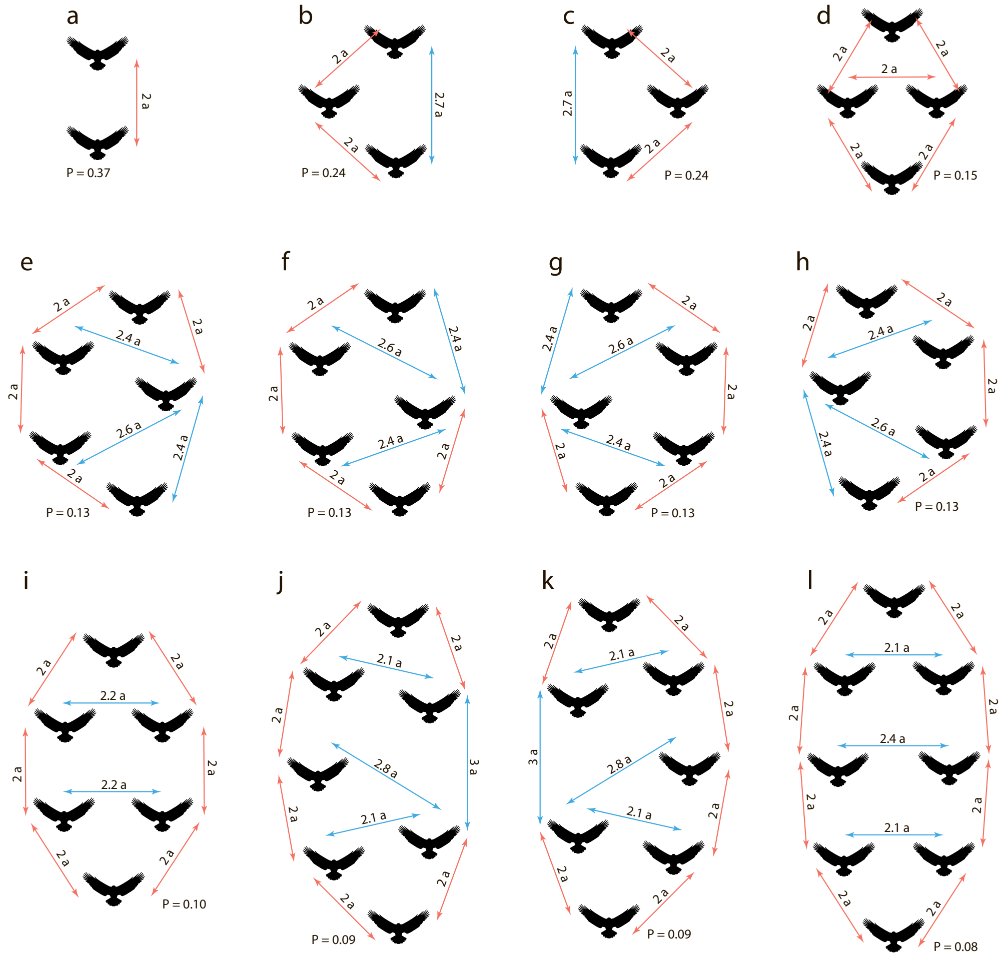

FIG. 11: Optimal configurations for a group of 2-8 agents moving with the same velocity. The results are obtained by the direct minimization of the total dissipation power. The average dissipated power per agent is indicated in units of the power dissipated by a solely moving agent.
